# Phenylpropanoids Are Connected to Cell Wall Fortification and Stress Tolerance in Avocado Somatic Embryogenesis

**DOI:** 10.3390/ijms21165679

**Published:** 2020-08-08

**Authors:** Carol A. Olivares-García, Martín Mata-Rosas, Carolina Peña-Montes, Francisco Quiroz-Figueroa, Aldo Segura-Cabrera, Laura M. Shannon, Victor M. Loyola-Vargas, Juan L. Monribot-Villanueva, Jose M. Elizalde-Contreras, Enrique Ibarra-Laclette, Mónica Ramirez-Vázquez, José A. Guerrero-Analco, Eliel Ruiz-May

**Affiliations:** 1 Red de Manejo Biotecnológico de Recursos, Instituto de Ecología A. C., Cluster BioMimic^®^, Carretera Antigua a Coatepec 351, Congregación el Haya, Xalapa, Veracruz CP 91073, Mexico; caro_aol@hotmail.com (C.A.O.-G.); martin.mata@inecol.mx (M.M.-R.); 2 Tecnológico Nacional de México, Instituto Tecnológico de Veracruz, Unidad de Investigación y Desarrollo en Alimentos, Veracruz CP 91897, Mexico; 3 Instituto Politécnico Nacional, Centro Interdisciplinario de Investigación para el Desarrollo Integral Regional-Unidad Sinaloa, Boulevard Juan de Dios Bátiz Paredes # 250, Col. San Joachin, Guasave, Sinaloa 81101, Mexico; fquirozf@hotmail.com; 4 European Molecular Biology Laboratory, European Bioinformatics Institute, Wellcome Genome Campus, Hinxton, Cambridgeshire CB10 1SD, UK; asegura@ebi.ac.uk; 5 Department of Horticultural Science, University of Minnesota, Saint Paul, MN 55108, USA; lmshannon@umn.edu; 6 Unidad de Bioquímica y Biología Molecular de Plantas, Centro de Investigación Científica de Yucatán, Mérida, Yucatán CP 97205, Mexico; vmloyola@cicy.mx; 7 Red de Estudios Moleculares Avanzados, Instituto de Ecología A. C., Cluster BioMimic^®^, Carretera Antigua a Coatepec 351, Congregación el Haya, Xalapa, Veracruz CP 91073, Mexico; juan.monribot@inecol.mx (J.L.M.-V.); jose.elizalde@inecol.mx (J.M.E.-C.); enrique.ibarra@inecol.mx (E.I.-L.); monica.ramirez@inecol.mx (M.R.-V.); joseantonio.guerrero@inecol.mx (J.A.G.-A.)

**Keywords:** plant cell wall, embryogenic cultures, metabolomics, phenolic compounds, proteomics

## Abstract

Somatic embryogenesis (SE) is a valuable model for understanding the mechanism of plant embryogenesis and a tool for the mass production of plants. However, establishing SE in avocado has been complicated due to the very low efficiency of embryo induction and plant regeneration. To understand the molecular foundation of the SE induction and development in avocado, we compared embryogenic (EC) and non-embryogenic (NEC) cultures of two avocado varieties using proteomic and metabolomic approaches. Although Criollo and Hass EC exhibited similarities in the proteome and metabolome profile, in general, we observed a more active phenylpropanoid pathway in EC than NEC. This pathway is associated with the tolerance of stress responses, probably through the reinforcement of the cell wall and flavonoid production. We could corroborate that particular polyphenolics compounds, including *p*-coumaric acid and *t*-ferulic acid, stimulated the production of somatic embryos in avocado. Exogen phenolic compounds were associated with the modification of the content of endogenous polyphenolic and the induction of the production of the putative auxin-a, adenosine, cellulose and 1,26-hexacosanediol-diferulate. We suggest that in EC of avocado, there is an enhanced phenylpropanoid metabolism for the production of the building blocks of lignin and flavonoid compounds having a role in cell wall reinforcement for tolerating stress response. Data are available at ProteomeXchange with the identifier PXD019705.

## 1. Introduction

Avocado (*Persea americana* Mill.) is a crop of great economic importance. However, avocado production is threatened by different agents, mainly fungal phytopathogens, including *Colletotrichum* species, *Phytophthora cinnamomi*, and *Euwallacea kuroshio* [1]. The losses caused by these diseases run into millions of dollars and severely affect the health of the trees, production, and international trade of avocado [2]. There have been large-scale efforts to breed disease-resistant avocados [3,4]. However, these efforts have been stymied by long breeding cycles and the challenges of controlling pollination under dichogamy. Implementation of biotechnological approaches, such as in vitro cultures, offers the possibility of the mass production of avocado. So far, the most promising tool for the in vitro generation of avocado is somatic embryogenesis [5].

Although somatic embryogenesis (SE) has been studied for several decades and considerable effort has been invested in establishing an efficient in vitro propagation system for avocado, the efficiency of somatic embryo conversion into plants remains low to non-existent [2,6,7,8]. It has been assumed that this is primarily due to a failure of somatic embryos to mature [6,9,10,11]. Attempts to improve the maturation of avocado somatic embryos using culture media components have failed [9,12,13]. These studies were limited by the paucity of molecular and biochemical information available about SE in avocado, not only at the maturation stage, but also at each prior stage of SE development, including the induction of embryogenic potency (EP), transdifferentiation, and germination [14]. Although the failure to mature of the somatic embryos is the most visible indication of unsuccessful SE, the problems leading to this deficit may occur earlier in development. Avocado somatic embryos that can proceed through the maturation process displayed higher proportions of proteins associated with stress response [14]. Studies suggested that reactive oxygen species (ROS) homeostasis and growth regulator signal pathways are pivotal orchestrators of molecular signaling during SE induction [15].

There is limited information about the molecular and biochemical processes governing the induction and development of SE in avocado. In different species, epigenetic modifications have been suggested as modulators of morphogenetic properties of in vitro cultures [16,17,18,19,20,21,22,23,24,25]. Furthermore, the phenylpropanoid pathway (PP) has been implicated in SE [26]. Embryogenic cultures in sugar beet and sandalwood have a fortified cell wall with a higher proportion of phenolic metabolites and lignin deposition as compared to non-embryogenic cultures [16,27]. However, overaccumulation of other phenolic compounds has displayed a negative effect on the establishment of SE in some species [22,28,29,30,31]. Thus, highly coordinated biochemical regulation of the PP may dictate the proper metabolic flow to orchestrate the induction or repression of SE. The recent publication of the avocado genome provides an extraordinary tool to unravel the secrets of SE in this plant species [32]. However, detailed proteomic and metabolomic studies in avocado are also needed to determine the key molecular players of the induction and progression of SE.

Despite the great importance of establishing a successful in vitro avocado propagation protocol, so far, there has been no success. Therefore, we took a step back and focused on the comparative proteomics between embryogenic (EC) and non-embryogenic callus (NEC) from the Hass and Criollo avocado varieties. Previous studies suggested this is a crucial comparison that could lead to the identification of the molecular players and increasing the current understanding of the establishment of EP in avocado cultures. In our experiments, the EC cultures exhibited a higher accumulation of proteins associated with phenylpropanoid metabolism, flavonoids, cell wall, and stress-related processes compared to NEC. In addition, the EC treated with polyphenolic compounds produced an increased number of embryos as compared to the control treatments. The increase in the efficiency of the SE was associated with the tight regulation of stress/growth regulator signal pathways, where PP plays an intricate role.

## 2. Results

### 2.1. Establishment of Non-Embryogenic and Embryogenic Cultures of Hass and Criollo Avocado

**Figure 1:**
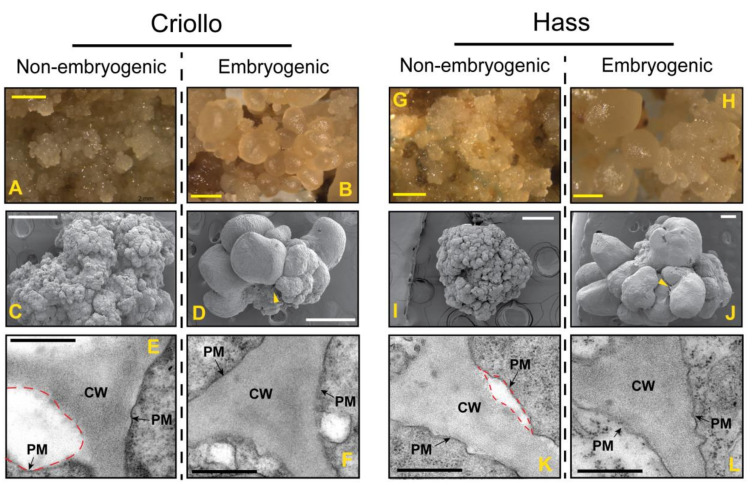
Phenotypes of non-embryogenic (NEC) and embryogenic (EC) cultures of avocado. Light microscopy (**A**,**B**,**G**,**H**), scan electron microscopy (SEM, **C**,**D**,**I**,**J**) and transmission electron microscopy micrographs (TEM, **E**,**F**,**K**,**L**) display contrasting differences between EC (**B**,**D**,**F**,**H**,**J**,**L**) and NEC (**A**,**C**,**E**,**G**,**I**,**K**) both in Criollo (**A**–**F**) and Hass (**G**–**L**) cultures. Yellow scale bars in (**A**), white scale bars in (**B**), and black scale bars in (**C**) denote 1 mm, 250 µm, and 500 nm, respectively. Yellow arrowhead indicates the extracellular matrix surface (EMSN) in (**D**,**J**). PM and CW indicate in (**C**) plasma membrane and cell wall, respectively. Dash red lines in (**C**) specified detachments of the PM from the cell wall.

Avocado embryogenic cultures were induced from immature zygotic embryo explants. In our study, we could induce direct SE in only 7% explants cultured in Murashige and Skoog medium supplemented with 0.1 mg L^−1^ picloram (MSP). This response was the same for both varieties. These successful cultures were then repeatedly subcultured in the same medium to develop embryogenic callus (EC). Only ~6% of the resulting callus lost embryogenic competence and became non-embryogenic callus (NEC, Figure 1), which is a well-known physiological feature of avocado cultures [2]. Light microscopy and SEM observations revealed that NEC is a white translucent friable mass (Figure 1A). On the other hand, pale yellow colored EC showed well-organized structures, including somatic embryos at an early globular stage (Figure 1B). The ECs also exhibited a layer resembling the extracellular matrix surface network (EMSN) observed in the EC of other plant species (Figure 1D,J, arrowhead). Close observation with TEM exhibited sharp differences in the cell walls between EC (Figure 1F,L) and NEC (Figure 1E,K). EC has a well-defined cell wall adjacent to the plasma membrane, while NEC displayed several detachments of the cell wall from the plasma membrane (Figure 1E,K, red dash lines).

### 2.2. Comparative Proteomics: Embryogenic vs. Non-Embryogenic Cultures

**Figure 2:**
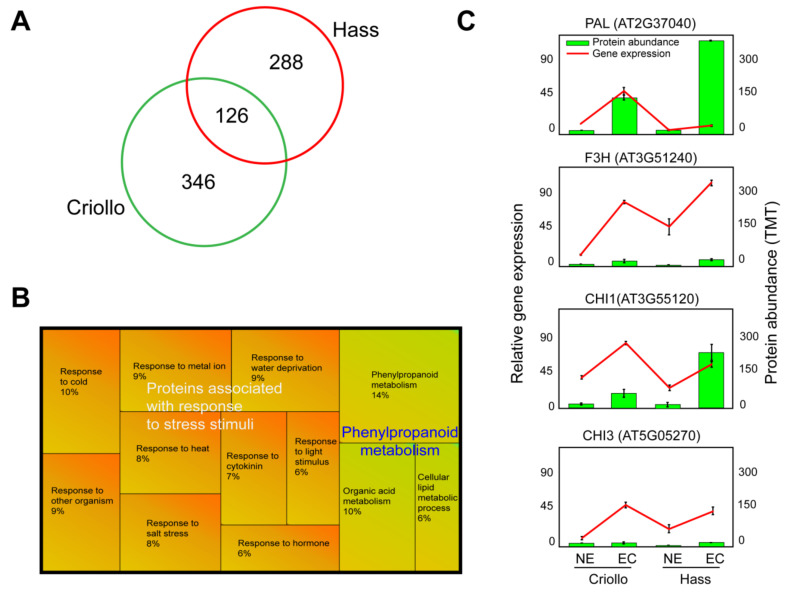
Differentially accumulated proteins identified by comparing embryogenic (EC) vs. non-embryogenic (NEC) cultures from Hass and Criollo avocado varieties. (**A**) The Venn diagram displays the number of differentially expressed proteins identified in Hass and Criollo cultures by comparing EC vs. NEC. (**B**) Core proteome representation-based gene ontology enrichment and clustering of biological process annotation (Data S2, **C**). Correlation between relative gene expression and protein abundance. The gene expression was normalized using Rubisco as an internal control. Points denote the mean fold expression as compared to the control ± three replicates of EC and NEC cultures of avocado cultivars “Hass” and “Criollo”. Phenylalanine ammonia-lyase 1 (*PAL*, AT2G37040), flavanone 3-hydroxylase (*F3H*, naringenin 2_oxoglutarate 3-dioxygenase, AT3G51240), Chalcone flavonone isomerase 1 (*CHI1*, AT3G55120), probable chalcone-flavonone isomerase 3 (*CHI3*, AT5G05270).

Our proteomic pipeline consisted of a peptide labeled with TMT6plex and synchronous precursor selection (SPS) MS3 (Figure S1). We could identify 1999 proteins in Hass samples while 1842 Criollo, among which 414 and 472 differentially expressed proteins by comparing EC and NEC in Hass and Criollo cultures, respectively (Figure 2A, Data S1, Data S2). Of those, 126 differentially accumulated proteins were identified in both varieties and indicated as core proteome in the following sections. Gene ontology enrichment and clustering based on biological processes highlighted in the core proteome two main groups of proteins, including those associated with the response to stress stimuli and phenylpropanoid metabolism (Figure 2B). We were able to identify well-known stress-related proteins, which were overaccumulated in EC compared to NEC (Table S1, Figure S2). Furthermore, the analysis of differentially accumulated proteins in both avocado varieties exhibited more proteins associated with stress stimuli in Criollo compared to Hass in vitro cultures (Table S1, Figure S3).

The core proteome exhibited well-annotated PP enzymes (Figure 2B). For instance, the phenylalanine ammonia-lyase 1 (*PAL1*) was accumulated in both Criollo and Hass EC compared to NEC, detecting significant values in Hass (Figure 2C). Gene expression of *PAL1* corroborates the significant activation of *PAL* in Criollo, while in Hass EC, *PAL1* was slightly upregulated compared to NEC (Figure 2C). In addition, a cytochrome P450, family 71, subfamily B, polypeptide 35 (CYP7135), peroxidase 52 (POX52), 4-coumarate: CoA ligase 1 (4CL1), caffeoyl-CoA *O*-methyltransferase 1 (CCoAOMT1), flavone 3’-*O*-methyltransferase 1 (OMT1) and leucoanthocyanidin dioxygenase (LDOX) were overaccumulated in Hass and Criollo EC compared to NEC (Table S1, Figure S2). The probable caffeoyl-CoA *O*-methyltransferase (CCoAOMT) was accumulated in Hass EC, while in Criollo EC, it displayed contrasting values. Aldehyde dehydrogenase family 2-member C4 (ALH2C4) exhibited a similar pattern of accumulation as that of CCoAOMT. In addition, the probable cinnamyl alcohol dehydrogenase 9 (CAD9) was accumulated in NEC compared to EC (Table S1, Figure S2). In addition to PP metabolism, proteins associated with the flavonoid biosynthetic process were identified in a higher proportion in EC than NEC. These proteins include the chalcone-flavanone isomerases (CHI1 and CHI3) and flavanone 3-hydroxylase (F3H, Figure 2C).

The upregulation of gene expression of *CHI1*, *CHI3*, and *F3H* in EC compared to NEC underpinned our proteomics data (Figure 2C). In addition, the UDP-glycosyltransferase superfamily protein with an annotation of quercetin 3-*O*-glucosyltransferase (GO: 0080043) and quercetin 7-*O*-glucosyltransferase transferase activity (GO: 0080044) was overaccumulated in EC (Figure S2). Untargeted metabolomics, followed by an OPLS-DA, exhibited the overaccumulation of putative polyphenolics such as naringenin, flavanone, and epicatechin 3’O- glucuronide in EC (Figure S4).

Proteins related to PP metabolism were also mainly accumulated in either Hass or Criollo varieties (Table S1, Figures S2 and S3). In Hass EC, we were able to determine the overaccumulation of two cytochrome P450 family proteins (C3’H, CYP98A3, and CYP71A22), annotated with the phenylpropanoid biosynthetic process (GO: 0009699), coumarin biosynthetic process (GO: 0009805), flavonoid biosynthetic process (GO: 0009813) and lignin biosynthetic process (GO: 0009809). Two additional cytochrome P450 family proteins (CYP93D1 and CYP94D2) were identified in a higher proportion in EC compared to NEC, which were annotated with the secondary metabolite biosynthetic process (GO: 0044550). In addition, we observed the overaccumulation of cinnamate-4-hydroxylase (C4H, CYP73A5) and UDP-glucosyl transferase 73B5 annotated with the molecular function of GO: 0080043, GO: 0080044 and flavonol 3-*O*-glucosyltransferase activity (GO: 0047893). In Criollo EC, we could identify the PAL2 in a higher proportion than in NEC. A similar pattern of overaccumulation was observed with the peroxidase 72 (POX72) associated with the lignin biosynthetic process, UDP-glucosyl transferase 85A2, with the molecular function of quercetin 3-*O*-glucosyltransferase and quercetin 7-*O*-glucosyltransferase transferase activity as well as UDP-glycosyltransferase superfamily protein associated with the molecular function of lignin biosynthetic process. In contrast, proteins related to the L-phenylalanine catabolic process (Figure S3), such as *p*-hydroxyphenylpyruvate dioxygenase and tyrosine decarboxylase 1, were overaccumulated in Criollo NEC compared to EC. 

Manual analysis of proteomic data also provided additional information on proteins associated with the cell wall biogenesis of avocado Hass and Criollo cultures. For example, the following proteins were identified in higher proportion in EC than NEC: probable pectinesterase/pectinesterase inhibitor 17, cellulose synthase-like E1, pectin lyase-like superfamily protein, probable glucan endo-1,3-β-glucosidases, and peroxidases were identified in higher proportion in EC than NEC (Table S1).

### 2.3. Phenolic Metabolites Improve the Production of Somatic Embryos in Hass and Criollo Avocado Embryogenetic Cultures

Our proteomic and untargeted metabolomic data suggested the activation of the phenylpropanoid pathway in avocado embryogenic cultures. Therefore, we carried out dose-response experiments by exposing EC to exponential concentrations (1, 10, 100, and 1000 µm) of specific polyphenolic compounds for 14 or 28 days. In our experiments, we included *trans*-ferulic acid (*t*-FA) due to its essential role in cell wall rigidity and the formation of other important organic compounds. We also selected *p*-coumaric acid (*p*-CA), a direct precursor of *p*-coumaroyl-CoA, and a branching point for the biosynthesis of flavonoids, monolignols and several other compounds [33]. We selected *p*-hydroxybenzoic acid (PHBA), associated with the final product of the β-oxidative pathway [34].

**Figure 3:**
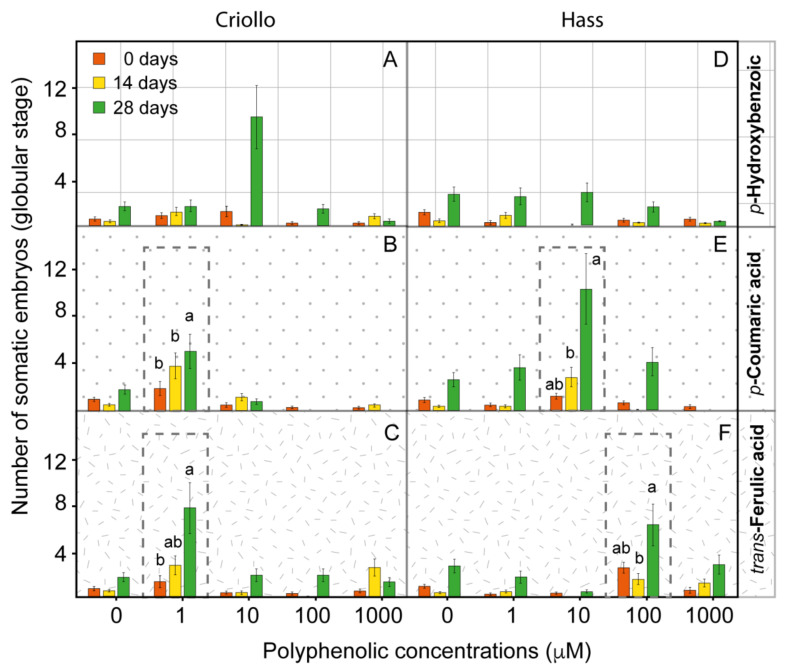
Effect of polyphenolics in embryogenic cultures during 28 days of dose-response treatments. The Criollo (**A**–**C**) and Hass (**D**–**F**) cultures were treated with 1, 10, 100, and 1,000 µm of *p*-hydroxybenzoic acid (**A**,**D**), *p*-coumaric acid (**B**,**E**), and *trans*-ferulic acid (**C**,**F**). The effect of polyphenolics was determined based on the number of newly formed globular embryos per plate. A general linear model (GLM) was applied with default settings to determine the statistical significance between treatments. Different letters (a, b and ab) represent a significant difference among treatments (*p* < 0.05) marked with dash lines.

Criollo EC treated with 10 µM PHBA exhibited a significant improvement in embryo production after 28 days (Figure 3A). In contrast, PHBA did not show a significant effect on the production of embryos in Hass EC (Figure 3B). In addition, *p*-CA enhanced the production of embryos in both Criollo and Hass cultures treated for 14 and 28 days with a concentration of 1 and 10 µm, respectively (Figure 3B,E). In both Criollo and Hass EC, the improvement in embryo production was significant after 28 days of treatment. Cultures treated with *t*-FA exhibited a similar boost of embryo production with a concentration of 1 and 100 µm in both cultures, observing significant values at 28 days (Figure 3C,F). A visual analysis of the appearance of the cultures corroborates the higher number of embryos in EC treated with the compounds, as mentioned earlier. In some cases, we could observe dark regions in treated tissues, especially those treated with *t*-FA, which could suggest phenolization of the tissues (Figure S5A,B). Surprisingly, embryos treated with *p*-CA and *t*-FA exhibited minimal *PAL1* gene expression after 24 h (Figure S6).

### 2.4. The Improvement of the Production of Embryos in Avocado EC Is Associated with the Alteration of the Endogenous Content of Polyphenolic Compounds

The PP is at the crossroad of plant growth, structural support, biotic, and abiotic stress. Stress stimuli are among the main driving force of the induction of SE [21]. Consequently, exposing EC to these metabolites might alter the metabolome during the overproduction of somatic embryos. To corroborate our hypothesis, we carried out a target metabolomics approach. We analyzed a short time of treatments, including 6 and 12 h, as well as extended periods of 14 (335 h) and 28 days (672 h, Figure S3).

We could determine a complete alteration of the endogenous content of several polyphenolics in EC treated with *p*-CA and *t*-FA for short times (Table 1, Table S2). The most noticeable change was associated with the sharp reduction in the content of *p*-CA, quercetin 3,4’-di-*O*-glucoside, *t*-FA, and vanillin (VA) in Criollo EC treated with 1 µM *p*-CA after 6 h compared to the control (Table 1). From 6 to 12 h, we could observe a general reduction in all polyphenolic compounds analyzed. However, after 14 days, the content of *p*-CA and PHBA was higher in control than treated EC with 1 µM *p*-CA and t-FA (Table 1). In contrast, PHBA acid showed an exponential overaccumulation after 28 days in samples exposed to 1 µM *p*-CA (Table 1). The PHBA and sinapic acid (SA) exhibited a slight increase in samples treated with 1 µM *t*-FA after 28 days of exposition.

The early effect of 10 µM *p*-CA in Hass EC included the drastic reduction in the content of quercetin 3,4’-di-*O*-glucoside, *t*-FA, and SA (Table 1). In contrast, *trans*-cinnamic acid (t-CA) exhibited a substantial increase in its content after 6 h of exposition. After that, quercetin 3, 4’-di-*O*-glucoside, *p*-CA, *t*-FA, and SA were detected in higher concentrations in control samples than EC treated with 10 µM *p*-CA after 12 h. The *t*-FA was the major polyphenolic detected at the last time of analysis in samples treated with 10 µM *p*-CA. The early effect of 100 µM *t*-FA in Hass EC was in contrast with previous analysis comprising the marked overaccumulation of VA, *p*-CA, quercetin 3,4’-di-*O*-glucoside, naringin, and *t*-CA compared to control sample (Table 1). The content of these polyphenolics exhibited a continuous reduction during the time of exposition while, *t*-FA showed a sharp increase after 14 days of analysis, and the content PHBA showed a slight increase after 28 days in tissues exposed to 100 µM *t*-FA in comparison with the control sample.

### 2.5. Untargeted Metabolomics Provides New Clues Related to the Effect of P-Coumaric and Trans-Ferulic Acid on the Improvement of Embryo Production in Avocado

**Figure 4:**
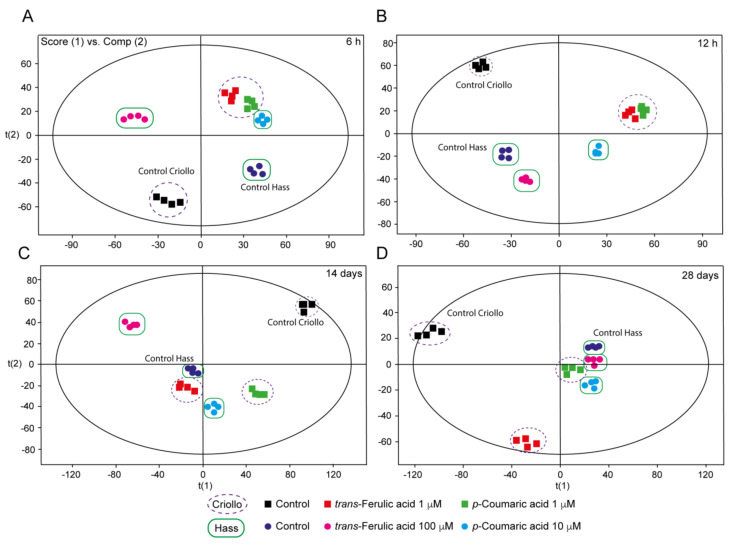
Principal component analysis (PCA)-mediated grouping of Criollo and Hass EC treated with *p*-coumaric acid and *trans*-ferulic acid over 6 h (**A**), 12 h (**B**), 14 days (**C**) and 28 days (**D**). We used the exact mass spectrum fingerprints to carry out the principal component analysis.

The principal component analysis (PCA) of our results exhibited a significant effect of 1 µM *p*-CA and *t*-FA on Criollo EC metabolome profile compared to control samples, throughout the time of exposition (Figure 4). After 12 h, the metabolic profile of Criollo EC treated with both polyphenolics was different compared with the control but similar between each treatment. After 14 and 28 days, the callus treated with 1 µM *p*-CA or *t*-FA and control samples exhibited different metabolic signatures (Figure 4C,D). Hass EC treated with 10 µM *p*-CA and 100 µM *t*-FA exhibited different metabolome profiles versus the control within 6 h (Figure 4A). After 12 h, only Hass EC treated with 10 µM *p*-CA showed a different profile in comparison to control samples (Figure 4B). In contrast, after 14 days of exposition samples treated with 100 µM, *t*-FA showed an entirely different metabolome signature compared to the control (Figure 4C). After 28 days of exposition, neither 10 µM *p*-CA nor 100 µM *t*-FA affected the metabolome profile of Hass EC, while Criollo EC treated with 1 µM *t*-FA exhibited an entirely different metabolome profiled compared to control sample (Figure 4D).

To determine possible molecular markers associated with the improvement of the proliferation of somatic embryos in EC, we focused on scrutinizing which metabolites are overaccumulated after 12 h and 28 days of treatment with *t*-FA 100 µM in Hass, and *t*-FA 1 µM in Criollo EC. 

**Figure 5:**
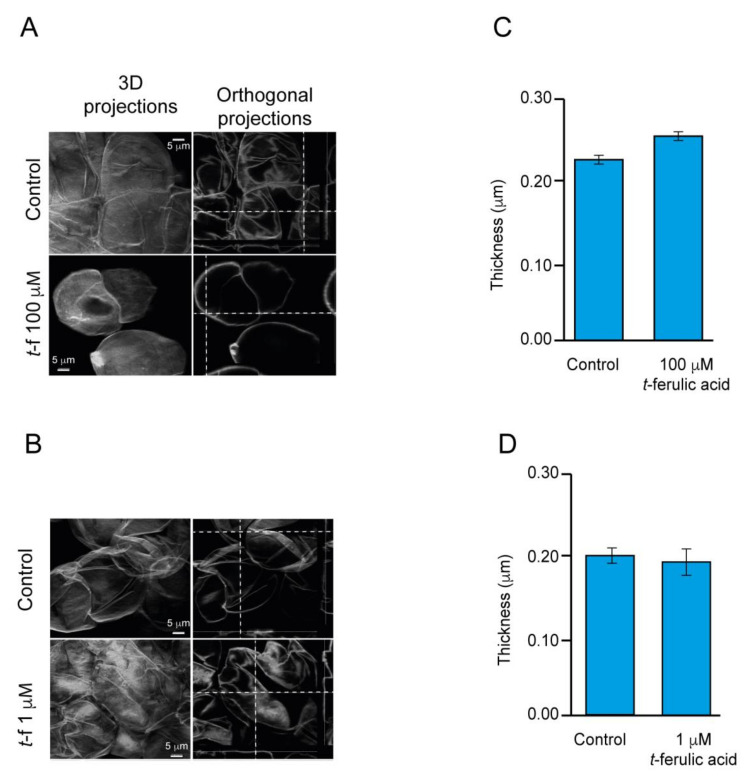
Determination of cell wall thickness during 12 h of treatment with ferulic acid in avocado Hass embryos. Three-dimensional projection base confocal microscopy analysis, intersections of dotted lines indicate the angle of analysis (**A**,**B**). The thicknesses of the cell wall were measured considering 30 orthogonal projections and the statistical analysis was made by R (**C**,**D**).

We were able to determine the overaccumulation of a putative auxin-a (FDB017851) and adenosine (FDB003554) after 12 h of treatment with both polyphenolics in the two avocado varieties (Table S3). Furthermore, after 28 days, Hass cultivar exhibited the overaccumulation of putative cellulose (FDB001182), while in Criollo, we could determine the putative 1,26-hexacosanediol-diferulate (FDB002683). Afterward, we carried out a microscopy study based on super-resolution confocal images to identify some modification at the cell wall reinforcement level. Our approach was associated with calcofluor labeling to highlight the cell wall, which allowed us to measure the thickness of it. Our study suggests that there is a slight thickening of the cell wall of embryos of avocado Hass varieties after 12 h of treatment with 100 µM t-FA (Figure 5A,C). Cell wall embryos treated with 1 µM *t*-FA did not exhibit significant differences compared to the control sample (Figure 5B,D). Furthermore, statistical analysis verified the significant thickening of the cell wall compared with the control samples (Figure 5C).

## 3. Discussion

### 3.1. Stress Regulation and Cell Wall Fortification a Common Feature of Embryogenic Callus and Possible Association with the Improvement of Embryogenesis

Previous studies have suggested that in vitro cultures face multiple types of stress during the acquisition of EP [21,35,36]. Our results indicate that NEC may face even more adverse conditions than EC. The cellular and molecular response to stress differ between avocado varieties (Table S1, Figures S2 and S3). To make our analysis more precise, we mainly focused on differential proteins that were differentially accumulated in the EC and NEC of both Criollo and Hass varieties in two independent studies (Core proteome, Figure 2, Figure S1). The core proteome in this study could be related to responses to stimuli, including responses to cold (GO: 0009409), water deprivation (GO: 0009414), light stimulus, metal ion (GO: 0010038), salt stress (GO: 0009651), heat (GO: 0009408), cytokinin (GO: 0009735), hormone (GO: 0009725) and other organisms (GO: 0051707, Figure 2B, Data S2). 

**Figure 6:**
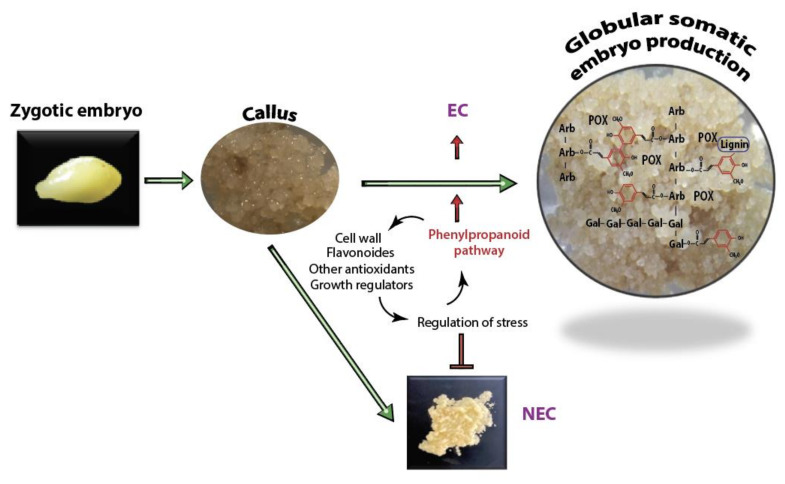
A model of cell wall reinforcement and regulation of stress condition mediated by the phenylpropane pathways and growth regulators in avocado somatic embryogenesis. In dicots, ferulic acid is bound to pectic polysaccharides, including the C-2 hydroxyl group of arabinofuranose (Arb) and C-6 hydroxyl group of galactopyranose (Gal). Peroxidases (POX) catalyze the oxidation of ferulic acid, leading the formation of ferulic acid dehydrodimers. The depiction of ferulic acid in the cell wall modified from Mathew and Abrahan [43].

There are several documented strategies of plant cells to overcome adverse external conditions, including the reinforcement of their cell walls and over-production of antioxidant metabolites [37,38]. Some of the aforementioned strategies may prevail in avocado embryogenic cultures (Figure 6). Evidence suggests that such stress response is critical in the establishment of SE. For example, peroxidases (POXs) are potential markers of EP acquisition across species [39,40]. The ROS-induced activity of POXs is the primary mechanism involved in wall remodeling during stress. POXs are proposed to crosslink cell wall glycoproteins such as extensins, facilitating arabinoxylans crosslinking through ferulic acid and promoting the formation of diferulic acid crosslinks between lignin molecules [41]. The higher number of extracellular POXs (Table S1) identified in ECs as compared to NEC points to a better biochemical response to oxidative stress, perhaps in part through cell wall reinforcement (Figure 1F,L). In addition, across species, the EMS is known to associate with arabinogalactans-proteins, hydroxyproline-rich glycoproteins, and pectin epitopes [42]. The presence of an EMSN and well-fortified cell wall in ECs (Figure 1D,J) suggests that these cell wall-associated proteins may play a vital role during the induction and establishment of SE in avocado cultures. Reinforcement of the cell wall with phenolic acid metabolites and lignin deposition has been indicated as a common feature of EC and organogenesis [16,27].

In EC, the up-regulation of cinnamic acid 4-hydroxylases (C4H, CYP73A5), which catalyzes the conversion of t-CA to *p*-CA, provides the first step toward the biosynthesis lignin monomers (Table S1). In addition, CCoAOMT1 identified in higher proportion in EC than NEC contributes to the production of feruloyl-CoA and sinapoyl-CoA, which are related to the synthesis of feruloylated polysaccharides and was directly implicated in cell wall strengthening [44]. In addition, C3’H (CYP98A3) overaccumulation in Hass EC (Table S1) is associated with the 3’-hydroxylation of *p*-coumaric esters of shikimic/quinic acids forming lignin monomers [45,46,47]. The complete repression of C3’H leads to cell wall alteration and reduction in lignin deposition, which in turn affects the cell wall expansion and plant growth [47]. This shows that C3’H could contribute to cell wall reinforcement during the formation of embryos in the early stage of SE. Furthermore, overaccumulation of POX72 and other related lignin proteins underpin the lignin monomer biosynthesis in Criollo EC (Figure 6). 

The up-regulation of flavonoid-related enzymes in EC, including chalcone-flavanone isomerases, naringenin, 2-oxoglutarate 3-dioxygenase and UDP-glycosyltransferase superfamily provides evidence of the activation of this branch of the phenylpropanoids in EC. As flavonoids are ROS scavengers, the overaccumulation of proteins linked to the biosynthesis of these molecules, for example, the overproduction of naringenin, flavanone, and epicatechin 3’*O*-glucuronide in EC (Figure S4), could indicate the contribution of these ROS scavengers to the maintenance of the ROS balance in EC [48,49].

### 3.2. The Improvement of the Production of Somatic Embryos by Polyphenolics Is Linked to the General Reduction in the Level of Endogenous Phenolic Compounds

Our proteomic data strongly suggest the activation of the PP in avocado EC. As previously mentioned, our analysis showed the activation of two main branches of the PP, including lignin and flavonoid biosynthesis. We hypothesized that exposing specific polyphenolics might increase the rate of embryo generation in EC based on our results of comparative proteomics between EC and NEC Criollo and Hass avocado varieties. We could determine that a lower concentration of 1 µM *p*-CA and *t*-FA significantly improved the rate of production of somatic embryos in Criollo EC. In comparison, 10 µM *p*-CA and 100 µM *t*-FA are better for Hass EC (Table 1). 

We could determine that treating Criollo and Hass EC with 1–10 µM *p*-CA and 1 µM *t*-FA reduced the production of several polyphenolic metabolites after 6 h of treatment. In contrast, exposing Hass EC to 100 µM *t*-FA exhibited contrasting patterns, as observed with the above-mentioned treatments after 6 h. Furthermore, Criollo and Hass EC presented the overaccumulation of PHBA and *t*-FA after 28 days of treatment, respectively. We should keep in mind that the dynamic of the endogenous content of polyphenolics in tissue cultures depends on several factors, such as the concentration and ratio of growth regulators, response to stress factors and grade of differentiation of tissues and organs [50,51,52,53].

In a previous study, a callus treated with cytokinin displayed a drastic reduction in *p*-CA, sinapic, *t*-FA, *t*-CA, and CA. In addition, a significant induction of hydroxybenzoic acid derivatives such as PHBA, MHBA, and vanillic acids was observed in callus cultures [52]. Cytokinin negatively regulates the expression of *PAL* genes [49]. On the other hand, the cyclic AMP molecule is involved in the activation of PAL [54]. The cyclic AMP was drastically accumulated in Criollo EC treated with *t*-FA 1 µM for 12 h (Table S2). The studies above strongly suggest an interconnection between growth regulators and the phenylpropanoid pathway. This assumption is supported by a previous report where naringenin and *cis*-cinnamic acid (c-CA, conversion of *t*-CA by light) were suggested as negative regulators of auxin transport [55]. Recent studies indicated that auxin-regulated plant growth is fine-tuned by early steps in phenylpropanoid biosynthesis, particularly *t*-CA, and its derivative enhances auxin signaling and promotes auxin-dependent leaf expansion in Arabidopsis [56]. In our targeted metabolomics study, we putatively detected the overaccumulation of Auxin A and purines in EC exposed with *t*-FA during the first 12 h in both Criollo and Hass varieties. We do not know the molecular function of the putative auxin-a in the SE of avocado. However, auxin signals are probably transduced through cAMP, but further evidence is needed to corroborate this assumption [57]. Auxin is an essential plant hormone for SE induction and the cellular differentiation process [58,59]. Ashinara et al. [59] noticed that an active utilization of purines during the early phases of SE might be required for the proliferation and cell division of the embryogenic tissue in the presence of auxin. The possible presence of these compounds could explain the proliferation of somatic embryos during the treatments with polyphenolic compounds. Further study may provide clues associated with the functionality of these molecules in avocado SE.

The PP, as a crossroad of several biological processes, is tightly regulated at several levels. For example, PAL, the first committed enzyme in the PP, is regulated at several levels, including the transcriptional regulation of *PAL* genes and posttranscriptional modifications. The activity of *PAL* is metabolically feedback regulated by particular biosynthetic intermediates or chemical signals [49]. In our study, we visualize the significant upregulation of *PAL1* gene expression in both Criollo and Hass EC compared to NEC (Figure 2C). However, in EC treated with *p*-CA and *t*-FA, the PAL1 gene expression was barely observed after 24 h (Figure S6). Previous studies in *Phaseolus vulgaris* cell suspension cultures showed that the exogenous application of t-CA negatively regulated the enzymatic activity of PAL activity and *PAL* gene transcription [60,61]. Our result and others suggest a negative regulation of *PAL* gene expression. Our target metabolomic analysis, showing a general reduction in endogenous content of polyphenolics, is supported by studies from different plant species where PAL activity was shown to be inhibited by *t*-CA, *p*-CA, PHBA, *O*-chlorocinnamate and other related compounds [62,63,64]. The negative feedback regulation of *PAL* might redirect carbon flow, as observed after *C4H* inhibition, which was associated with the production of namoylmalate and overproduction of salicylic acid in Arabidopsis plants and elicited *Nicotiana tabacum* cv Bright Yellow cell suspension culture, respectively [65,66]. The negative regulation of *PAL* gene expression in Criollo EC and Hass EC was associated with the significant overaccumulation of PHBA and *t*-FA, respectively, after 28 days of treatment with *p*-CA (Table 1).

### 3.3. Change in Metabolic Flow May Explains the Positive Association between Polyphenolics and the Overproliferation of Avocado Somatic Embryos

It is noteworthy to mention that previous studies have indicated that phenolic compounds including *t*-CA, PHBA, vanillyl benzyl ether (VBE), 4-[(phenyl methoxy) methyl] phenol, caffeic acid, and chlorogenic acid, inhibit SE [22,31]. Although the precise inhibition mechanism of these secondary metabolites is unknown, overaccumulation of phenolic metabolites in the culture media of dense in vitro cultures abolished the induction and establishment of SE. Cvikrová et al. [67] reported a negative correlation between the mitotic activity and the content of hydroxycinnamic acids in the alfalfa cell suspension. PHBA, which was overaccumulated in Criollo EC treated with 1 µM *p-*CA after 28 days, has been associated with the repression of SE induction in *Larix leptolepis*, *Coffea canephora* and *Daucus carota* [22,31]. However, it is possible that PHBA and other related phenolic metabolites became inactive while associated with the cell wall. In avocado culture, the induction of somatic embryos by *p*-CA and *t*-FA may comprise redirection of the metabolic flow from the synthesis of polyphenolics to the production of the building blocks of lignin and flavonoid compounds having a role in cell wall reinforcement and ROS scavenging, respectively. The general reduction in endogenous content of polyphenolics in EC treated with *p*-CA and *t*-FA during the first 12 h supports our assumption (Table 1). Previous studies have suggested that an adverse effect of *p*-CA on PAL1 might change the metabolic flow toward the production of well-known lignin constituents [68,69]. 

Previous studies suggested the crucial role of cell wall reinforcement in the early stages of SE [70,71]. In addition, TEM showed a well-defined cell wall of EC compared to NEC in avocado cultures (Figure 1E,F,K,L), the reinforcement of which can be associated with both the cross-linking of feruloyl-polysaccharides or oxidative coupling of lignin precursors [72,73], which in part may explain the slight increase in cell wall thickness detected with confocal microscopy in Hass avocado treated with *t*-FA 100 µM for 12 h (Figure 5).

## 4. Materials and Methods

### 4.1. Chemical Reagents

Solvents used for the extraction and analysis of phytochemicals (methanol, isopropanol, acetonitrile, water and formic acid) were LC-MS grade (67-56-1, 67-63-0, 75-05-8, 7732-18-5, 85178, respectively, Sigma-Aldrich, (St. Louis, MO, USA). Authentic standards for mangiferin, (+)-catechin, quercetin-3-D-galactoside, quercetin, gallic acid, (-)-epicatechin and quercetin-3-glucoside (M3547, 43412, 83388, Q4951, G7384, 1753, 16654, respectively, Sigma-Aldrich, St. Louis, MO, USA) were used. Kaempferol-3-*O*-glucoside (90242), 4-hydroxy-benzoic acid (99-96-7), caffeic acid (6034 S), 4-coumaric acid (6031 A), ferulic acid (6077 A), quercetin 3, 4-di-*O*-glucoside (1347 S), quercetin 3-D-*O*-galactoside (1027 S) standards were purchased from Extrasynthese (https://www.extrasynthese.com/). All reagents used for proteomic analysis were purchased from Sigma-Aldrich (St. Louis, MO, USA), except as otherwise specified in the corresponding section.

### 4.2. Establishment of Embryogenic Cultures

Immature fruits (5–10 mm) of avocado varieties “Hass” and “Criollo” were collected and ECs of both varieties were established from immature zygotic embryos [9]. A small proportion of the resulting ECs lost embryogenic competence after two cycles of the subculture; these cultures were labeled as NEC for this study. The media used to subculture both kinds of callus included MS (Murashige and Skoog) major and minor salt [74], sucrose 30 g L^−1^, thiamine HCl 4 mg L^−1^, myo-inositol 100 mg L^−1^ and picloram 0.41 µM; this medium is named MSP [10]. The pH of the culture medium was adjusted to 5.7 before adding gellan gum 3 g L^−1^ (Cat. No. 71010-52-1, Caisson Labs, Smithfield, UT, USA) (https://caissonlabs.com/), and autoclaving at 1.5 kg cm^−2^ at 121 °C for 15 min. Under aseptic conditions, aliquots of media (20 mL) were dispensed onto sterile plastic Petri dishes (100 × 15 mm). The cultures were incubated in darkness at 25 ± 1 °C. The explants were subcultured every two weeks on the same culture medium over seven months.

### 4.3. Protein Extraction

Proteins were obtained from 0.5 g of frozen NEC and EC collected from seven-month-old cultures and after two weeks of subculturing. The in vitro cultures were first ground with liquid nitrogen in a mortar with a pestle. The powder was suspended in three volumes of phosphate buffer 100 mM (pH 7.0) containing SDS 2%, NaCl 150 mM, and 20 µL g^−1^ of protease inhibitor cocktail (L P8849, Sigma-Aldrich). The mixtures were homogenized with a tissue homogenizer (Tissue-Tearor™, BioSpec Products, Inc., Bartlesville, OK, USA) and centrifuged at 10,000× *g* for 45 min at 25 °C. Afterwards, the supernatants were recovered and kept at −80 °C for future proteomic analysis. The protein assay was carried out with the BCA™ Assay Kit (23225, Pierce, Rockford, IL, USA) using bovine serum albumin (BSA) as a standard.

### 4.4. Proteomics Analysis

The general procedure of proteomic analysis, including protein digestion and isobaric labeling, has been published elsewhere [75]. Detailed information on proteomic analysis is presented in Appendix A, and particular modifications in the general procedures are summarized in this section.

### 4.5. Protein Digestion and Tandem Mass Tags (TMT) Labeling

We started with 100 µg of protein by reducing it with tris (2-carboxyethyl) phosphine (TCEP; 10 mM, C4706, Sigma Aldrich) and alkylating with iodoacetamide (IA, A3221 Sigma Aldrich). Then, proteins were digested with trypsin (V528A, Trypsin Gold, Promega, Madison, WI, USA) at a 1:30 (*w/w)* trypsin protein ratio for 16 h at 37 °C. Afterwards, additional freshly made trypsin was added in 1:60 (*w/w*) trypsin protein ratio for 4 h at 37 °C. Then, peptides were labeled with TMT6-plex reagents according to the manufacturer’s instructions (90066, Thermo Fisher Scientific, Rockford, IL, USA). The labels 126, 127 and 128 were used for NECs, while labels 129, 130 and 131 were used for ECs. Then, samples were pooled and fractionated using strong cation exchange (SCX) cartridges (60108-421, Thermo Scientific). Fractions were desalted with C_18_ cartridges and dried using a CentriVap (Labconco Kansas, MO, USA).

### 4.6. NanoLC-MS/MS Analysis and Synchronous Precursor Selection (SPS)-MS3 for TMT Analysis

Each reconstituted sample (5 µL) was injected into a nanoviper C_18_ trap column (3 µm, 75 µm × 2 cm, Dionex) at a flow rate (FR) of 3 µL min^−1^, and fractionated on an EASY spray C18 RSLC column (2 µm, 75 µm × 25 cm) adapted to a nanoLC (UltiMate 3000 RSLC system, Dionex). A 100 min gradient was used with an FR of 300 nL min^−1^ and two solvents (solvent A: 0.1% formic acid in water and solvent B: 0.1% formic acid in 90% acetonitrile). The gradient was set as follows: 10 min solvent A, 7–20% solvent B for 25 min, 20% solvent B for 15 min, 20–25% solvent B for 15 min, 25–95% solvent B for 20 min, and 8 min solvent A. Full MS scans in the Orbitrap analyzer (Orbitrap FusionTM TribidTM, Thermo-Fisher Scientific, San Jose, CA, USA) were carried out with: 120,000 of resolution (FWHM), scan range 350–1500 *m*/*z*, AGC of 2.0e5, maximum injection time of 50 ms, intensity threshold of 5 × 10^3^, dynamic exclusion 1 at 70 s, and 10 ppm mass tolerance. For MS2 analysis, the 20 most abundant MS1s were isolated with charge states set to 2–7. A precursor selection mass range of 400–1200 *m*/*z* was used, with a precursor ion exclusion width range of 18 to 5 *m*/*z*, and an isobaric tag loss TMT. MS3 spectra were acquired using synchronous precursor selection (SPS) with ten isolation notches, as previously described [75].

### 4.7. Data Analysis and Interpretation

Raw data were processed with Proteome Discoverer 2.1 (PD, Thermo Fisher Scientific, USA). The subsequent search was carried out with SEQUEST HT, MASCOT (version 2.4.1, Matrix Science), and AMANDA against the avocado proteins databases. Parameters in the search included full-tryptic protease specificity, two missed cleavage allowed. Static modifications covered carbamidomethylation of cysteine (+57.021 Da) and TMT 6-plex *N*-terminal/lysine residues (+229.163 Da). Dynamic modifications comprised methionine oxidation (+15.995 Da) and deamidation in asparagine/glutamine (+0.984 Da). We used for the TMT6-plex quantification method ± 10 ppm mass tolerance, highest confidence centroid, and a precursor co-isolation filter of 45%. Protein identification was carried out with tolerances of ±10 ppm and ±0.6 Da. Peptide hits were filtered for a maximum of 1% FDR using the Percolator algorithm. Functional annotation of proteins was carried out by Blast2Go software (https://www.blast2go.com/), and gene ontology (GO) enrichment was carried out by David bioinformatic source (https://david.ncifcrf.gov/) using *Arabidopsis* homologs. We used the REVIGO web server (http://revigo.irb.hr/) for GO clustering and visual representation of biological processes (Data S2).

### 4.8. Phytochemical Extraction

Fifty milligrams of EC and NEC samples were lyophilized in a freeze dryer (Freezone1, Labconco, Kansas, Missouri, USA) and suspended in 1 mL of methanol containing 0.1% formic acid and then homogenized with a tissue homogenizer (Tissue-Tearor™, BioSpec Products, Bartlesville, OK, USA) in a 1.5 mL centrifuge tube. Afterwards, the samples were placed in an ultrasonic bath (Cole-Parmer, Vernon Hills, IL, USA) for 45 min at 4 °C. Then, the mixtures were centrifuged at 3000× *g* for 15 min at 4 °C. The supernatant was split off for further analysis. Three biological replicates were analyzed for NEC and EC.

### 4.9. Determination and Quantification of Phenolic Compounds and Untargeted Metabolomics Analysis

Phenolics were identified and quantified using a UPLC system (Agilent, 1290, Santa Clara, CA, USA) coupled to the QqQ mass spectrometer (Agilent, 6460, Santa Clara, CA, USA) with a dynamic multiple reaction monitoring (dMRM) method for the searching for up to 60 compounds, as previously described by our research group [76]. In addition, untargeted metabolomics analysis using liquid chromatography and high-resolution mass spectrometry and orthogonal partial least square discriminant analysis (OPLS-DA) was carried out as previously reported [75]. Detailed information is shown in Appendix A. 

### 4.10. Stereoscope Analysis

Fresh tissue samples of CE and NEC of Hass and Criollo varieties were observed in a light stereoscope Leica S6D (Schweiz) with a 63× objective. The images were processed using LAS V4.12 software (http://www.leicamicrosystems.com).

### 4.11. Scanning Electron. Microscopy Preparation (SEM)

The samples from 7-month-old EC and NECs were fixed in glutaraldehyde 2.5% (pH 7.2) for 12 h. After fixation, the cultures were rinsed thrice in phosphate buffer (pH 7.2) and dehydrated in an ethanol series for 60 min each (40%, 50%, 60%, 70%, 80%, 90% to 100%). After ethanol dehydration, the samples were dried in a critical point drier (K 850, Quorum, West Chester, PA, USA) using liquid CO_2_. Samples were then attached to an aluminum stub with double-stick tape. The cultures were then gold coated in a sputter coater (Q150R, Quorum, Quorum Technologies Ltd., Lewes, UK) and observations were carried out in an FEI-Quanta250 FEG microscope (Czech Republic), operated at 5 Kv acceleration voltage.

### 4.12. Transmission Electron. Microscopy (TEM)

The samples were fixed overnight with 0.1 M ‘Sorensens’s buffer (pH 7.2) containing paraformaldehyde 2%, glutaraldehyde 1% and sucrose 0.8%. Afterwards, the samples were rinsed with the same buffer and post-fixed with OsO4 1% for 2 h. Thereafter, samples were dehydrated with different concentrations of ethanol for 10 min each (30, 50, 70, 96 and 100%). Then the samples were embedded in ‘Spurr’s resin (14300, Electron Microcopy Science, Hatfield, PA, USA) and polymerized at 60 °C for 24 h. Afterward, thin cross-sections of 70 nm were obtained with an Ultracut ultramicrotome (EM UC7, Leica Microsystem, Wetzlar, Germany) (http://www.leicamicrosystems.com) and mounted on copper grids and studied with a JEM 1400 Plus Transmission Electron Microscope (JEOL, Akishima, Tokyo, Japan).

### 4.13. Confocal Microscopy

Avocado somatic embryos for Confocal Laser Scanning Microscopy (CLSM, Mannheim, Germany) were fixed in 4% paraformaldehyde in phosphate-buffered saline, 0.2 M (pH 7.2; PBS). Samples were stained with 10 µL of calcofluor white (18909 Sigma-Aldrich, (St. Louis, MO, USA) for 10 min and rinsed in PBS for 5 min. Images were acquired with a Leica TCS-SP8+STED microscope (Leica Microsystems, Mannheim, Germany) using a plan-apochromat 63x (NA 1.40, oil) objective. Calcofluor-stained samples (cell wall) were recorded in the grey channel (425–500 nm emission; excitation 405 nm). Deconvolution images were processed with SVI Hygens professional software v.18.04.1 (Hilversun, The Netherlands).

### 4.14. Embryogenic Callus Treatments with Phenolic Compounds

Criollo EC was cultured in Petri dishes (100 × 15 mm) containing 25 mL of MSP medium supplements with *p*-hydroxybenzoic acid, *p*-coumaric acid (1 µM) or *trans*-ferulic acid for 28 d. At the same time, Hass EC was cultured for the same time in MSP medium containing either *p*CA 10 µM or *t*FA 100 µM. We sampled EC for 0, 6, 12, 24, 336 (14 d) and 672 h (28 d). The number of somatic globular embryos was visually recorded at 14 and 28 d of each treatment. The experiment consisted of five repetitions per treatment.

### 4.15. RNA Extraction and Quantitative PCR Assays (qPCR)

Total RNA was extracted using “Mini Kit Plant RNeasy” following the manufacturer’s instructions (74104, Qiagen, Hilden, Germany). Sequence-specific primers were designed for each gene. Arabidopsis genes homologs in avocado, including phenylalanine ammonia-lyase 1 (*PAL1*, AT2G37040), chalcone-flavanone isomerases (*CHI1*, AT5G05270 and *CHI*3, AT3G55120) and flavanone 3-hydroxylase (*F3H*, AT3G51240) were chosen. The primers were designed using Primer Express Software (version 3.0.1, IDT). Primer or probe sequences were selected to be complementary to an exon-exon junction, to ensure amplification from the cDNA template and not from genomic DNA. The transcript abundance of the genes in this study was analyzed by quantitative real-time PCR (qRT-PCR) with the StepOne™ Real-Time PCR System (Applied Biosystems, Foster City, CA, USA) using TaqMan assays. The gene expression values were normalized to Rubisco (accession No. AY337727) expression. The RT-PCR reaction was performed under the following conditions: 50 °C for 2 min, 10 min at 95 °C, followed by 40 cycles at 95 °C each 10 s, 60 °C for 1 min for denaturation, primer alignment, and amplification, respectively. Finally, qRT-PCR data were analyzed by the 2− ΔΔCt method [77]. Analyses were performed per triplicate. The statistical analysis was performed using one-way ANOVA (*p* < 0.05), followed by Tukey’s honestly significant test calculated at 5% levels of probability using the Statistical Software R^®^.

### 4.16. Statistical Analysis

Protein abundances were quantile, and log2 normalized. A linear model for microarrays data [78] approach with default setting was used to determine the statistical significance of differential protein expression between ECs and NECs for all the quantified proteins. Significantly differentially expressed proteins were defined as proteins identified with at least two peptides, which exhibited a 2-fold-change in EC to NEC ratio with a *p*-value smaller than 0.05. The statistical analysis of the endogenous content of phenolic compounds was carried out with a Mann–Whitney U-test (*n* = 3, *p* < 0.05).

## 5. Conclusions

Our proteomic-metabolomic approach provides insight into the improvement of SE in avocado. Our findings highlight the critical features of EC, including an active PP metabolism and cell wall fortification that might be linked with molecular features associated with embryogenic competence. Both ECs and NEC experience stress due to culture conditions, proteins associated with oxidation-reduction processes, and other stress response mechanisms were, however, present in a higher proportion in EC than NEC. This suggests that the ability to respond to stress is a hallmark of SE establishment in avocado. Our obtained evidence indicates that specific polyphenolics can induce the proliferation of embryos, which could be useful for the obtention of starting biological material during the establishment of an efficient pipeline for the improvement of embryo maturation/germination in the particular case of avocado SE.

## Figures and Tables

**Table 1:** The endogenous concentration of phenolic compounds (mg g^−1^ fresh weight) in embryogenic cultures of Criollo and Hass avocado treated with different concentrations of *p*-coumaric (1 and 10 µM) and *trans*-ferulic acid (10 and 100 µM) during 6 and 12 h, and 14 and 28 days. The analysis was carried out with dynamic multiple reaction monitoring (dMRM) study. Replicate values, error standard and statistic values are presented in Table S2. *p*-hydroxybenzoic acid (PHBA), vanillin (VA), *p*-coumaric acid (*p*-CA), *trans*-ferulic acid (*t*-FA), SA (Sinapic acid) and *trans*-cinnamic acid (*t*-CA). We indicated in bold and italic the values described in the text.

Cultivar Time	Treatment	PHBA	VA	*p*-CA	Quercetin 3,4’-di-*O*-glucoside	*t*-FA	SA	Naringin	*t*-CA
Criollo 0 h	0 µM Control	0.11 ± 0.01	0.11 ± 0.01	0.42 ± 0.01	0.02 ± 0.01	0.23 ± 0.01	0	0.07 ± 0.01	0.02 ± 0.01
	1 µM *p*-CA	0.11 ± 0.01	**0.11 ± 0.01**	**0.42 ± 0.01**	**0.02 ± 0.01**	**0.23 ± 0.01**	0	0.07 ± 0.01	0.02 ± 0.01
	1 µM *t*-FA	0.11 ± 0.01	0.11 ± 0.01	0.42 ± 0.01	0.02 ± 0.01	0.23 ± 0.01	0	0.07 ± 0.01	0.02 ± 0.01
Hass 0 h	0 µM Control	0.21 ± 0.01	0.03 ± 0.03	0.66 ± 0.02	0.01 ± 0.01	0.09 ± 0.01	0	0	0.04 ± 0.01
	10 µM *p*-CA	0.21 ± 0.01	0.03 ± 0.03	0.66 ± 0.02	0.01 ± 0.01	0.09 ± 0.01	0	0	0.04 ± 0.01
	100 µM *t*-FA	0.21 ± 0.01	0.03 ± 0.03	0.66 ± 0.02	0.01 ± 0.01	0.09 ± 0.01	0	0	0.04 ± 0.01
Criollo 6 h	0 µM Control	0.58 ± 0.01 ***	0.22 ± 0.01 ***	0.85 ± 0.01 ***	1.66 ± 0.07 ***	1.12 ± 0.01 ***	0.7 ± 0.01 ***	0.34 ± 0.01 ***	0.06 ± 0.01 ^**^
	1 µM *p*-CA	0.64 ± 0.05 ***	0.06 ± 0.01 **	0.29 ± 0.01 **	0 *	0.12 ± 0.01 **	0 **	0 **	0.4 ± 0.01 ***
	1 µM *t*-FA	0.38 ± 0.01 **	0.05 ± 0.01 **	0.18 ± 0.01 **	0.03 ± 0.04 **	0.28 ± 0.01 **	0 **	0 **	0.11 ± 0.01 ***
Hass 6 h	0 µM Control	0.29 ± 0.01 *	0.06 ± 0.01 *	0.62 ± 0.01 *	0.33 ± 0.05 *	1 ± 0.01 **	0.86 ± 0.02 ***	0.06 ± 0.01 *	0.03 ± 0 *
	10 µM *p*-CA	0.25 ± 0.01 *	0.05 ± 0.001 *	0.7 ± 0.01 **	0.02 ± 0.03 **	0.13 ± 0.01 *	0 *	0 *	0.29 ± 0.01 *
	100 µM *t*-FA	0.32 ± 0.01 *	0.24 ± 0.01 ***	1.39 ± 0.01 ***	0.62 ± 0.02 ***	10.6 ± 0.22 ***	0.64 ± 0.02 **	0.18 ± 0.02 ***	0.98 ± 0.02 ***
Criollo 12 h	0 µM Control	0.21 ± 0.01 **	0.15 ± 0.01 ***	0.61 ± 0.01 ***	0.62 ± 0.04 ***	0.52 ± 0.01 ***	0.72 ± 0.02 ***	0.05 ± 0.01 ***	0.02 ± 0.01 **
	1 µM *p*-CA	0.25 ± 0.01 ***	0.06 ± 0.01 **	0.46 ± 0.01 **	0 **	0.26 ± 0.01 **	0 **	0 ***	0.15 ± 0.01 ***
	1 µM *t*-FA	0.18 ± 0 *	0.04 ± 0.01 *	0.18 ± 0.01 *	0 **	0.12 ± 0.01 *	0 **	0 ***	0.03 ± 0.01 **
Hass 12 h	0 µM Control	0.6 ± 0.01 ***	0.11 ± 0.01 *	0.94 ± 0.02 ***	0.18 ± 0.02 ***	0.86 ± 0.07 ***	0.42 ± 0.01 ***	0.21 ± 0.01 ***	0.12 ± 0.01 **
	10 µM *p*-CA	0.2 ± 0 *	0.03 ± 0.001 *	0.24 ± 0.01 *	0.04 ± 0.01 **	0.13 ± 0.01 *	0 *	0 *	0.08 ± 0.01 *
	100 µM *t*-FA	0.4 ± 0 *	0.17 ± 0.01 ***	0.48 ± 0.01 *	0.05 ± 0.04 **	0.39 ± 0.01 *	0 *	0.22 ± 0.01 ***	0.08 ± 0.01 *
Criollo 14 days	0 µM Control	1.12 ± 0.02 ***	0.06 ± 0.001 ***	1.28 ± 0.02 ***	0.3 ± 0.01 ***	0.23 ± 0.01 *	0.03 ± 0.02 ***	0 ***	0.01 ± 0.02 ***
	1 µM *p*-CA	0.09 ± 0.001 *	0.05 ± 0.01 **	0.39 ± 0.01 *	0 **	0.28 ± 0.02 **	0 **	0 ***	0.01 ± 0.02 ***
	1 µM *t*-FA	0.12 ± 0.01 **	0.02 ± 0.01 *	1.18 ± 0.08 **	0 **	0.56 ± 0.03 ***	0.02 ± 0.01 *****	0 ***	0.02 ± 0.01 ***
Hass 14 days	0 µM Control	0.09 ± 0.01 *	0.03 ± 0.01 ***	0.69 ± 0.03 ***	0 *	0.4 ± 0.01 **	0.01 ± 0.01 **	0 ***	0.02 ± 0.01 ***
	10 µM *p*-CA	0.11 ± 0.01 **	0.03 ± 0.01 ***	0.24 ± 0.02 *	0.06 ± 0.01 **	0.55 ± 0.03 **	0.1 ± 0.02 ***	0 ***	0.01 ± 0.01 **
	100 µM *t*-FA	0 *	0 *	0 *	0 *	7.06 ± 3.63 ***	0 **	0 ***	0 *
Criollo 28 days	0 µM Control	0.62 ± 0.01 *	0.05 ± 0.01 **	0.96 ± 0 ***	0 ***	0.72 ± 0.01 ***	0.23 ± 0.01 **	0 ***	0.5 ± 0 ***
	1 µM *p*-CA	3.56 ± 0.06 ***	0 *	0.05 ± 0.04 *	0 ***	0 *	0 *	0 ***	0 *
	1 µM *t*-FA	1.35 ± 0.02 **	0.08 ± 0 ***	0.74 ± 0.01 **	0.02 ± 0.04 ***	0.12 ± 0.10 **	1.55 ± 0.12 ***	0 ***	0.03 ± 0 **
Hass 28 days	0 µM Control	0.4 ± 0 *	0.05 ± 0 **	1.14 ± 0.01 ***	0 ***	0.78 ± 0.01 **	0.04 ± 0.01 ***	0 ***	0.01 ± 0 ***
	10 µM *p*-CA	1.36 ± 0.03 ***	0 *	0.02 ± 0.02 *	0 ***	7.75 ± 4.43 ***	0 *	0 ***	0 **
	100 µM *t*-FA	1.12 ± 0.01 **	0.03 ± 0 *	0.16 ± 0.01 *	0 ***	0.08 ± 0 **	0.01 ± 0.01 **	0 ***	0 **

1. A general linear model (GLM) for metabolomics data was applied with default settings to determine. the statistical significance between treatments. 2. Data are mean ± standard deviation reported in mµ g^−1^ of dried EC. 3. *, **, ***, ***** represent a significant difference among treatments (*p* < 0.05).

## Supplementary Materials

Supplementary materials can be found at https://www.mdpi.com/1422-0067/21/16/5679/s1. Figure S1: Workflow of proteomic studies carried out in avocado in vitro cultures, Figure S2: Volcano plots of differential proteins identified in Criollo (a) and Hass (b) avocado embryogenic (EC) and non-embryogenic cultures (NEC), Figure S3: Gene ontology enrichment and clustering of biological processes annotation of proteins accurately identified as differential either in Criollo or Hass cultures, Figure S4: S-Plot (Hass NE_EC) of the OPLS-DA comparison between Hass EC and NEC, Figure S5: Visual analysis of Criollo (a) and Hass (b) embryogenic cultures treated with one, ten, 100 and 1000 µM of 4-hydroxybenzoic acid, *p*-coumaric acid, and *trans*-ferulic acid for 28 days, Figure S6: Quantitative real-time PCR validation of for genes involved in phenylpropanoids pathway in treatments with polyphenolic compounds, Table S1: Core proteome differentially identify in both Hass and Criollo EC and NEC (-), based on LIMMA (linear model for microarray data), Table S2: Statistical analysis of values associated with the endogenous content of polyphenolics (mg g−1 fresh weight) in embryogenic Criollo and Hass cultures treated with different concentrations of *p*-coumaric acid and *trans*-ferulic acid during six h, twelve h, 14 and 28 days, Table S3: Putative identification of compounds detected in embryogenic Criollo and Hass cultures treated with *p*-coumaric acid (1 µM) and *trans*-ferulic acid (100 µM) after 12 h and 28 days, Table S4: Primers designed for quantification of relative expression by qPCR, Data S1: Differential proteins identified in comparative analysis, using TMT 6-plex label reagents and synchronous precursor selection (SPS) MS3, in embryogenic (EC) and non-embryogenic (NEC) Hass avocado cultures, Data S2: Gene ontology (GO) enrichment of the core proteome, proteins particularly identified in Hass and Criollo varieties-based David Functional annotation Bioinformatics Microarray analysis (https://david.ncifcrf.gov/) using Arabidopsis homologs. We used the REVIGO web server (http://revigo. irb.hr/) for GO clustering and visual representation of biological processes.

## Abbreviations

ALH2C4	Aldehyde dehydrogenase family 2-member C4
*c*-CA	*cis*-cinnamic acid
*p*-CA	*p*-coumaric acid
*t*-CA	*trans*-cinnamic acid
CAD9	Cinnamyl alcohol dehydrogenase 9
BSA	Bovine serum albumin
CCoAOMT	Caffeoyl-CoA *O*-methyltransferase
CCoAOMT1	Caffeoyl-CoA *O*-methyltransferase 1
C4H/ CYP73A5	Cinnamic acid 4-hydroxylases
4CL1	4-coumarate: CoA ligase 1
EC	Embryogenic callus
EMSN	Extracellular matrix surface Network
EP	Embryogenic potency
*t*-FA	*trans*-ferulic acid
GLM	General linear model
LDOX	Leucoanthocyanidin dioxygenase
MS	Murashige and Skoog
MSP	Murashige and Skoog medium supplemented with 0.1 mg L^−1^ picloram
NEC	Non-embryogenic callus
OMT1	Flavone 3’-O-methyltransferase 1
PAL1	Phenylalanine ammonia-lyase 1
PCA	Principal component analysis
PHBA	*p*-hydroxybenzoic acid
POX52	Peroxidase 52
PP	Phenylpropanoid pathway
ROS	Reactive oxygen species
SA	Sinapic acid
SE	Somatic embryogenesis
TCEP	tris (2-carboxyethyl) phosphine
TMT	Tandem mass tags
VBE	Vanillyl benzyl ether
VA	Vanillin

## Appendix A

Detailed information of methods used for comparative proteomic analysis, targeted study of phenolics (dynamic multiple reaction monitoring, dMRM) and untargeted metabolomics using liquid chromatography and high-resolution mass spectrometry and orthogonal partial least square discriminant analysis (OPLS-DA).
