# Supplementary material for: Phenylpropanoids Are Connected to Cell Wall Fortification and Stress Tolerance in Avocado Somatic Embryogenesis
Source: Int J Mol Sci. 2020 Aug 8;21(16):5679. doi: 10.3390/ijms21165679 (PMC7460882; doi:10.3390/ijms21165679)
Supplement: Supplementary file 1 [file ijms-21-05679-s001.zip › Appendix A.docx]

**Appendix 1.** Detailed information about methods used for comparative proteomic analysis, targeted analysis of phenolics (dynamic multiple reaction monitoring, dMRM) and untargeted metabolomics using liquid chromatography and high-resolution mass spectrometry and orthogonal partial least square discriminant analysis (OPLS-DA).

*Trypsin digestion, TMT labeling, simple fractionation and desalting*

A 100 µg protein extract was reduced with tris (2-carboxyethyl) phosphine (10 mM; TCEP) for 45 min at 60 °C, alkylated with iodoacetamide (IA; 30 mM) for 1 h at 25 °C in the dark. The remnant of IA was quenched with 30 mM DTT for 10 min. Thereafter, proteins were precipitated with four volumes of ice-cold acetone overnight at -20 °C. Subsequently, proteins were obtained by centrifugation at 3000 x *g* for 30 min at 4 °C. The supernatant was discarded, and the resulting protein pellet washed twice with fresh cold- acetone. The same conditions were used for centrifugation. After the last spin, the supernatant was discarded and the pellet was dried at 25 °C for 15 min. Afterward, the protein pellet was resuspended in 100 μL of 50 mM triethylammonium bicarbonate (TEAM) and 0.1% SDS. Proteins were digested with trypsin (V528A, Trypsin Gold, Promega, Madison WI) at a 1:30 (w/w) trypsin protein ratio, for 16h at 37 °C. Thereafter, additional fresh made trypsin was added in 1:60 (w/w) trypsin protein ratio for 4h at 37 °C. Then, peptides were labeled with TMT6-plex reagents according to manufacturer's instructions (90066, Thermo Fisher Scientific, Rockford, IL). Our study included three biological replicates, the labels 126, 127 and 128 were used for NECs while labels 129, 130 and 131 were used for ECs. Labeled samples were pooled and fractionated using strong cation exchange (SCX) cartridges (cat. no. 60108-421, Thermo Scientific, Bellefonte, PA, USA). Briefly, SCX cartridges were equilibrated with buffer A, containing 5mM KH2PO4 buffer (pH 3.0) in 25% acetonitrile (ACN). Then samples were loaded on the SCX cartridge. Subsequently, peptides bound to the SCX resin were rinsed with buffer A. Then peptides were sequentially eluted from the cartridge with increasing KCl concentrations. Four fractions were collected including the flow through and three consecutive elutions with increasing concentrations of KCl (150, 250, and 500 mM). Subsequently, each fraction was desalted with C_18_ cartridges and dried using a CentriVap (Labconco Kansas, Missouri).

*NanoLC-MS/MS analysis*

Samples were analyzed with a nanoLC (UltiMate 3000 RSLC system, Dionex, Sunnyvale, CA) connected to an Orbitrap Fusion^TM^ Tribid^TM^ (Thermo-Fisher Scientific, San Jose, CA) mass spectrometer equipped with an “EASY Spray” nano ion source (Thermo-Fisher Scientific, San Jose, CA). Each reconstituted sample (5 µL) was injected into a nanoviper C_18_ trap column (3 µm, 75 µm X 2 cm, Dionex) at 3 µL min^-1^, and then fractionated on an EASY spray C18 RSLC column (2 µm, 75 µm x 25 cm) using a 100 min gradient with a flow rate (FR) of 300 nL min^-1^ and two solvents (solvent A: 0.1% formic acid in water and solvent B: 0.1% formic acid in 90% acetonitrile). The gradient was as set follows: 10 min solvent A, 7-20% solvent B for 25 min, 20% solvent B for 15 min, 20-25% solvent B for 15 min, 25-95% solvent B for 20 min, and 8 min solvent A. The MS was functioned in positive ion mode with nanospray voltage set at 3.5kV and source temperature at 280 °C. External calibrants included caffeine, Met-Arg-Phe-Ala (MRFA) and Ultramark 1621.

*Synchronous precursor selection (*SPS*)-*MS3 *for TMT analysis*

Full MS scans in the Orbitrap analyzer were carried out with: 120,000 (FWHM) of resolution, scan range 350-1500 m/z, AGC of 2.0e5, maximum injection time of 50 ms, intensity threshold of 5.0e3, dynamic exclusion 1 at 70 s, and 10 ppm mass tolerance. For MS2 analysis the 20 most abundant MS1 were isolated with charge states set to 2-7. CID fragmentation parameters included: 35% of collision energy, activation Q of 0.25, AGC of 1.0e4 and maximum injection time of 50 ms, a precursor selection mass range of 400-1200 m/z, precursor ion exclusion width range 18 m/z to 5 m/z, and isobaric tag loss TMT. Detection was conducted in the ion trap. Afterward, MS3 spectra were acquired as previously described [1] using SPS with 10 isolation notches. MS3 precursors were fragmented by HCD with 65% of collision energy and analyzed using the Orbitrap with a resolution of 60,000 at 120-500 m/z scan range, 2 m/z isolation window, 1.0e5 AGC, and maximum injection time of 120 ms with 1 microscan.

*Avocado in-house database assembly for proteomic analysis*

Unigenes derived from transcriptomics data available from both varieties, Hass [2] and “criollo” [3], were used to create our database. Each unigene was translated in the six possible reading frames. Those coding sequences which, once translated, generated the longest amino acids sequences, were selected as representative proteins from each unigene. Only “complete proteins” were used to construct theoretical mass spectra that were used on the analysis based on overlap or 'cross-correlation'. A protein was considered as complete when the amino acid sequences derived from their corresponding coding sequences represent at least 70% of the length from four of six homologous proteins identified by blast searches in closely related species such *Amborella trichopoda* or some other angiosperm species distributed in phylogenetic specific clades (Monocots: *Spirodela polyrhiza*; Eudicots: *Aquilegia coerulea*, *Solanum lycopersicum*, *Vitis vinifera* and *Arabidopsis thaliana*). The latest versions of these proteomes were downloaded from CoGe database (https://genomevolution.org/coge/). Blast search against UniProtKB/Swiss-Prot database was performed to retrieve UniProt annotation.

*Data analysis and interpretation*

Raw data were processed with Proteome Discoverer 2.1 (PD, Thermo Fisher Scientific Inc.). The subsequent search was carried out with SEQUEST HT [4], MASCOT (version 2.4.1, Matrix Science, Boston, MA) and AMANDA [5] against the avocado proteins databases, which was generated as we described above. Parameters in the search included full-tryptic protease specificity and two missed cleavage allowed. In addition, static modifications covered carbamidomethylation of cysteine (+57.021 Da) and TMT 6-plex N-terminal/lysine residues (+229.163 Da). Furthermore, dynamic modifications comprised methionine oxidation (+15.995 Da) and deamidation in asparagine/glutamine (+0.984 Da).

The TMT6-plex quantification method was carried out with the PD software. We used a ±10 ppm mass tolerance, highest confidence centroid, and a precursor co-isolation filter of 45%. For the SPS-MS3 method, the quantification was performed at the MS3 level. For protein-level reporting, protein grouping was enabled, and values were calculated from the median of all quantifiable peptide-to-spectrum matches (PSMs) for each group (master proteins).

Functional annotation of Master proteins was carried out by Blast2Go software (https://www.blast2go.com/), and functionally classified by David bioinformatic source (https://david.ncifcrf.gov/) using Arabidopsis homologs. We used REVIGO web server (http://revigo. irb.hr/) for the visual representation of functional classification.

*Detailed information about the determination and quantification of phenolic compounds by dynamic multiple reaction monitoring (dMRM).*

The analyses were carried out in a 1290 infinity Agilent ultra-high performance liquid chromatography (UPLC) system coupled to a 6460 Agilent triple quadrupole (QqQ) mass spectrometer with an Agilent, Zorbax SB-C18, 2.1 x 50 mm, 1.8 micron column. The mobile phases were water (A) and acetonitrile-water in a ratio of 9:1 (B), both with 0.1% formic acid at a flow rate of 0.1 Ml min^-1^. The gradient elution started from 1% B, then changed from 1 to 40% B over 40 min, then changed from 40 to 90% B over 2 min, then isocratic in 90% B for 2 min, then from 90 to 1% B over 2 min and finally isocratic in 1% B for 1 min. The column temperature was 40 ^°^C and 1 µL of sample was injected. The ESI source was operated in positive mode for (+)-catechin, (-)-epicatechin, mangiferin, quercetin-3-D-galactoside, quercetin-3-glucoside, kaempferol-3-*O*-glucoside and quercetin while gallic acid was determined in negative mode. The desolvation temperature was 300 ^°^C; N_2_ gas flow, 5 L min^-1^; nebulizer pressure, 45 psi; sheath gas temperature, 250 ^°^C; sheath gas flow, 11 L min^-1^; capillary voltage (positive and negative), 3500 V and nozzle voltage (positive and negative), 500 V. The dMRM transitions for each compound were searched in public databases as Metlin and corroborated experimentally in our laboratory. The precursor and product ions were considered as qualifier ion and the product ion was considered as the quantifier ion. The retention time for each compound was experimentally determined for gallic acid (3.8 min), (+)-catechin (11.2 min), (-)-epicatechin (14.48 min), mangiferin (15.18 min), quercetin-3-D-galactoside (20.6 min), quercetin-3-glucoside (21.19 min), kaempferol-3-*O*-glucoside (23.64 min) and quercetin (29.7 min). The cell accelerator voltage was 7 V for each compound. For quantification, a calibration curve with 10 concentrations points (0.5, 1, 3, 5, 7, 9, 11, 13, 15 and 17 µM) was prepared. Each calibration point was injected twice, and their respective areas were considered for a quadratic regression. The coefficient of determination was higher than 0.99 for each compound. The data was obtained with the Agilent MassHunter Workstation software (B.06.00). The transitions used were: mangiferin 423>302.8, (+)-catechin 291>138.9, quercetin-3-D-galactoside 465>302.9, quercetin-3-glucoside 465>303, kaempferol-3-*O*-glucoside 449>286.9, quercetin 302.9>153.1, gallic acid 169>125.2 and (-)-epicatechin 291>138.8. The fragmentor voltage used for all compounds was 100 V. The collision energy used was10 V except for quercetin (35 V).

*Untargeted metabolomics analysis using liquid chromatography and high-resolution mass spectrometry and orthogonal partial least square discriminant analysis (OPLS-DA)*

The chromatographic system was a Waters UPLC Class I coupled to a Synapt HDMi mass spectrometer. The chromatography was carried out on an Acquity BEH column (1.7 µm, 2.1 x 50 mm) with column and sample temperatures of 40 and 15 °C, respectively. The mobile phase consisted of (A) water and (B) acetonitrile, both with 0.1% of formic acid (SIGMA). The gradient conditions were 0-13 min linear gradient 1-0% B, 13-14 min 80% B isocratic, 14-15 min linear gradient 80-1% B (total run time 20 min). The flow rate was 0.3 mL min^-1^ and 2 µL of extract was injected. Mass spectrometric analysis was performed with an electrospray ionization source in positive mode. The capillary, sampling cone and source offset voltages were 3000, 40 and 80 V, respectively. The source temperature was 100 °C and the desolvation temperature was 20 °C. The desolvation gas flow was 600 L h^-1^ and the nebulizer pressure was 6.5 Bar. Leucine-enkephalin was used as the lock mass (556.2771, [M+H]^+^). The conditions used for MS analysis were: mass range 50-1200 Da, Function 1 CE, 6 V, function 2 CER 10-30 V, scan time 0.5 sec. Data were acquired and processed with MassLynx (version 4.1) and MarkerLynx (version 4.1) software, determining the discriminant chemical biomarkers through orthogonal partial least square discriminant analysis (OPLS-DA). Retention times and protonated masses were generated at a noise threshold of 10,000 counts and smoothing was applied. Pareto scaling was applied to generate the score plots. In the S-plots, the variables that contributed to discrimination between two groups were considered as potential biomarkers. Tentative identification was performed making reference to the public database Metlin (<https://metlin.scripps.edu/landing_page.php?pgcontent=mainPage>), considering the molecular ion exact mass and the fragmentation pattern for each compound.

References

1. McAlister, G.C., Nusinow, D.P., Jedrychowski, M.P., Wu¦êhr, M., Huttlin, E.L., Erickson, B.K., Rad, R., Haas, W., Gygi, S.P. MultiNotch MS3 enables accurate, sensitive, and multiplexed detection of differential expression across cancer cell line proteomes. *Anal. Chem.* **2014,** *86*, 7150-7158, https://doi.org/10.1021/ac502040v.

2. Kilaru, A., Cao, X., Dabbs, P.B., Sung, H.J., Rahman, M.M., Thrower, N., Zynda, G., Podicheti, R., Ibarra-Laclette, E., Herrera-Estrella, L. Oil biosynthesis in a basal angiosperm: transcriptome analysis of *Persea americana* mesocarp. *BMC Plant Biol.* **2015,** *15*, 203, https://doi.org/10.1186/s12870-015-0586-2.

3. Ibarra-Laclette, E., Méndez-Bravo, A., Pérez-Torres, C.A., Albert, V.A., Mockaitis, K., Kilaru, A., López-Gómez, R., Cervantes-Luevano, J.I., Herrera-Estrella, L. Deep sequencing of the Mexican avocado transcriptome, an ancient angiosperm with a high content of fatty acids. *BMC Genomics* **2015,** *16*, 599, 10.1186/s12864-015-1775-y.

4. Eng, J.K., McCormack, A.L., Yates, J.R. An approach to correlate tandem mass spectral data of peptides with amino acid sequences in a protein database. *J. Am. Soc. Mass Spectrom.* **1994,** *5*, 976-989, https://doi.org/10.1016/1044-0305(94)80016-2.

5. Dorfer, V., Pichler, P., Stranzl, T., Stadlmann, J., Taus, T., Winkler, S., Mechtler, K. MS Amanda, a Universal identification algorithm optimized for high accuracy tandem mass spectra. *J. Proteome Res.* **2014,** *13*, 3679-3684, https://doi.org/10.1021/pr500202e.
