# Supplementary material for: Phenylpropanoids Are Connected to Cell Wall Fortification and Stress Tolerance in Avocado Somatic Embryogenesis
Source: Int J Mol Sci. 2020 Aug 8;21(16):5679. doi: 10.3390/ijms21165679 (PMC7460882; doi:10.3390/ijms21165679)
Supplement: Supplementary file 1 [file ijms-21-05679-s001.zip › Supplemental_FigS1-S6_TableS1-S4.docx]

**
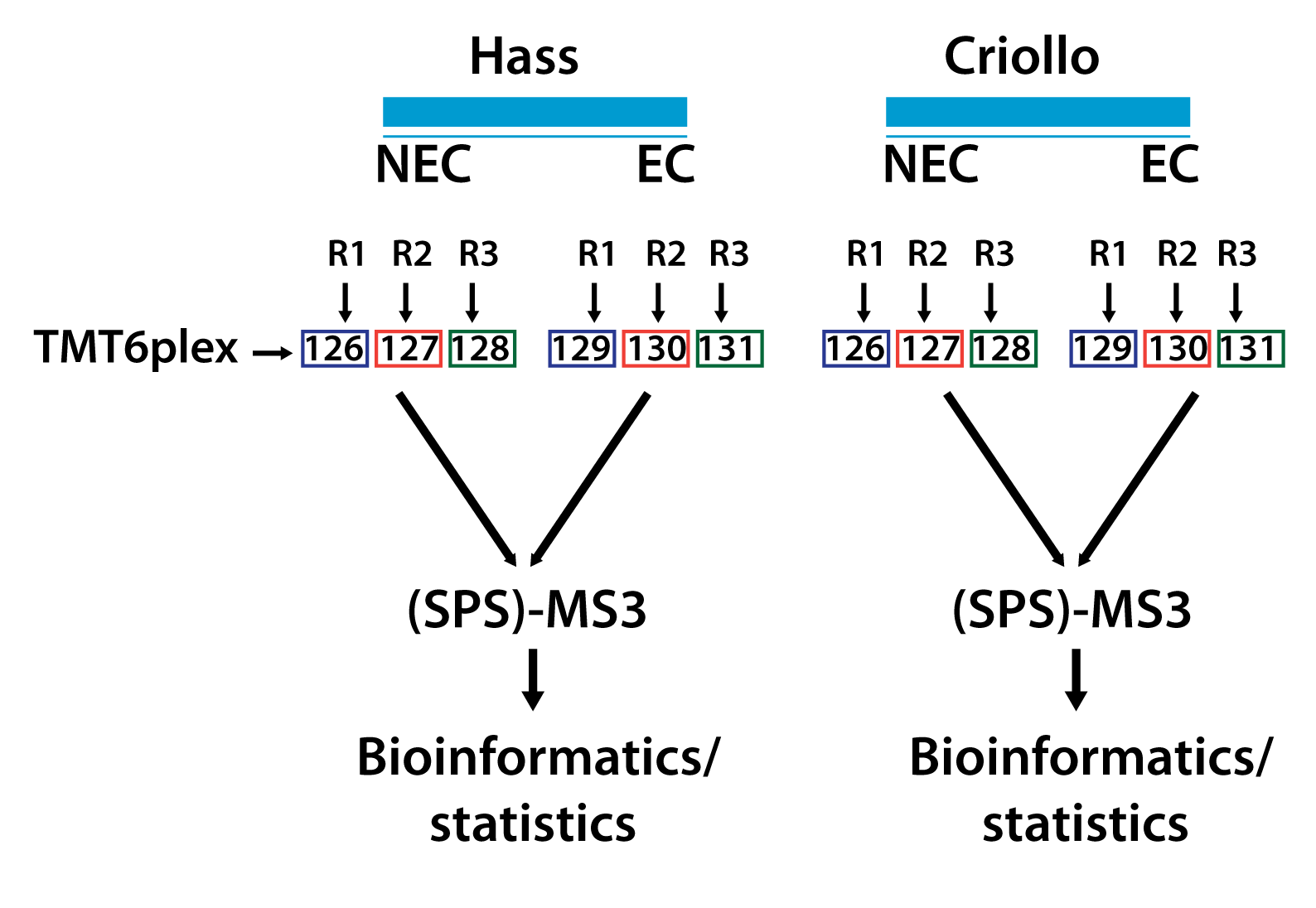
**

**Figure S1.** Workflow of proteomic studies carried out in avocado *in vitro* cultures. Hass and Criollo avocado cultures were analyzed independently. Three biological replicates were considered for proteomic analysis. Non-embryogenic cultures (NEC) were labeled with 126, 127 and 128 TMT tags and embryogenic cultures (EC) were labeled with 129, 130 and 131 TMT tags.

**Figure S2.** Volcano plots of differential proteins identified in Criollo (a) and Hass (b) avocado embryogenic (EC) and non-embryogenic cultures (NEC). Red dots indicate differential proteins with significant values (EC / NEC ratio > 2 and *P* < 0.05). Positive Log_2_FC indicates over-accumulation in EC and negative Log_2_FC indicates over-accumulation in NEC.

**
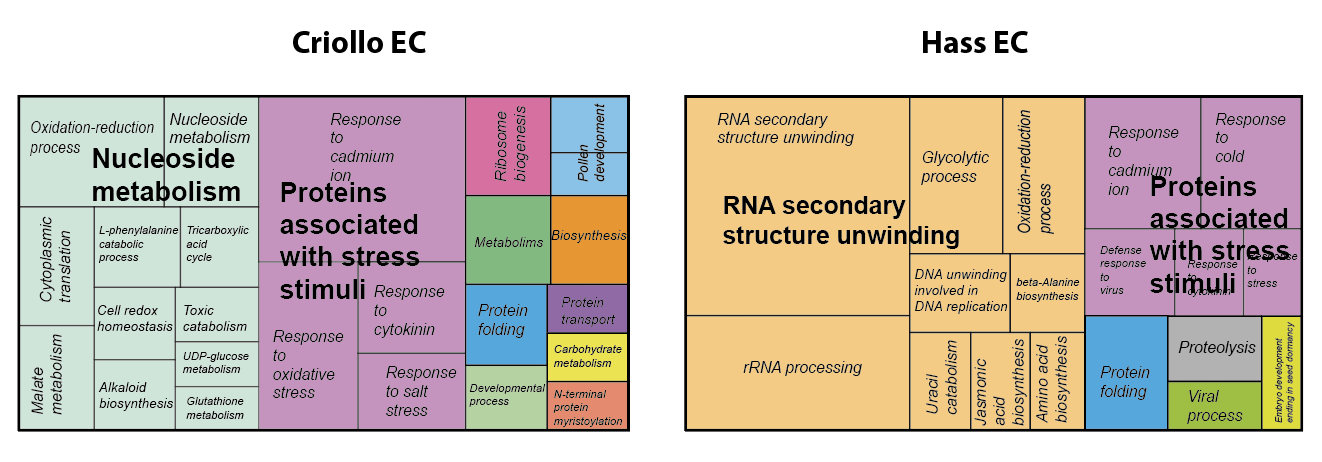
**

**Figure S3.** Gene ontology enrichment and clustering of biological processes annotation of proteins specifically identify as differential either in Criollo or Hass cultures.

**
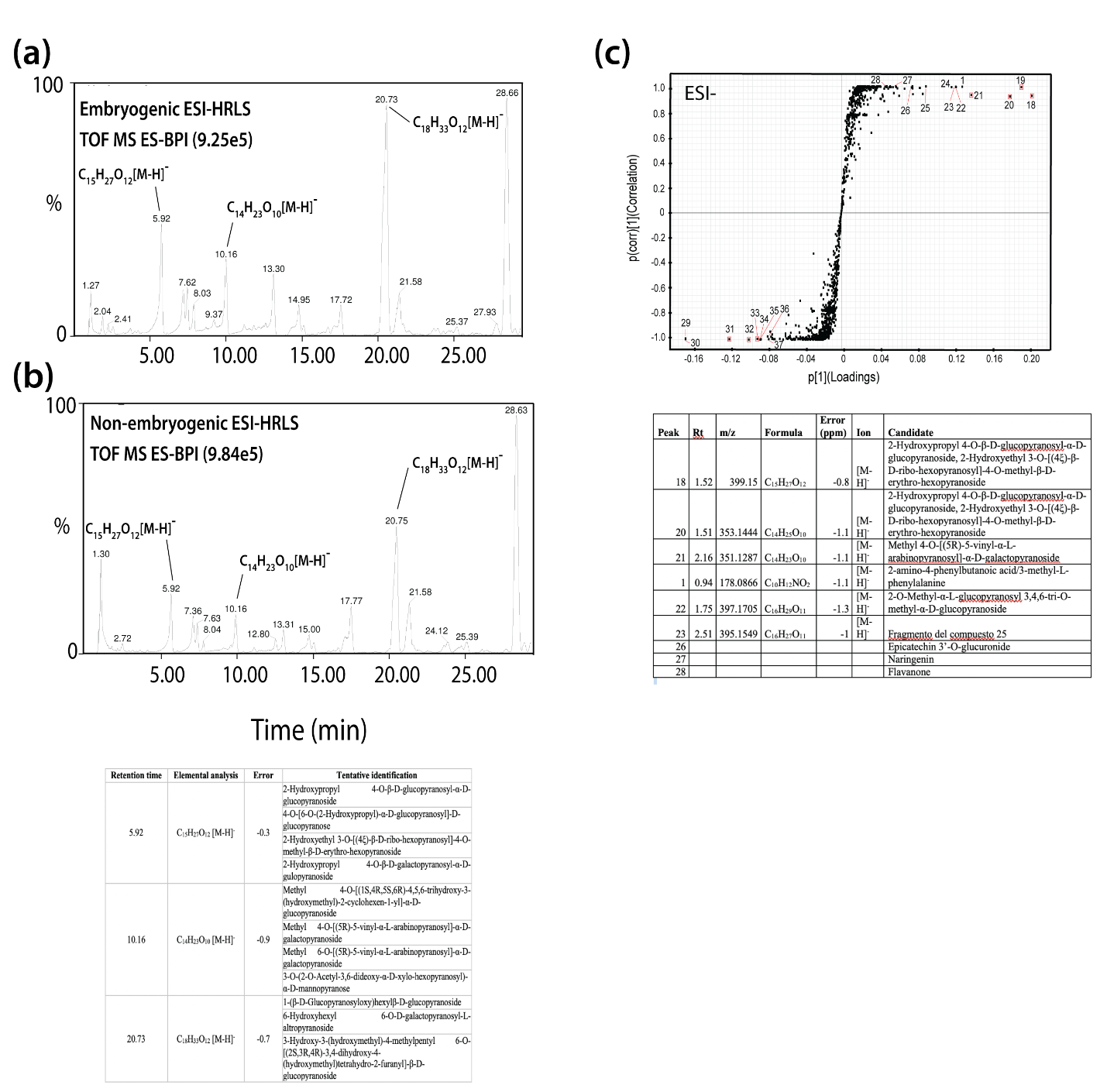
**

**Figure S4.** Tentative compounds identified by their MS spectral data obtained in a high-resolution mass spectrometer, computer elemental analysis and through their comparison with a natural occurring products database. (c) *S*-Plot (Hass NE_EC) of the OPLS-DA comparison between Hass EC and NE.

**
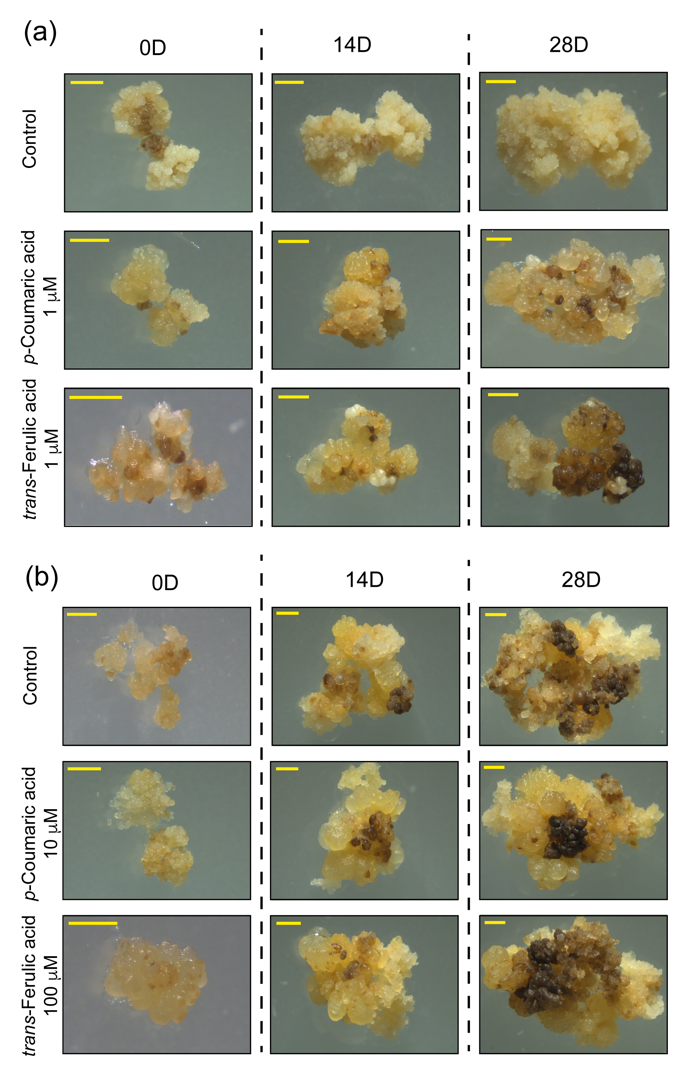
**

**Figure S5**. Visual analysis of Criollo (a) and Hass (b) embryogenic cultures treated with one, ten, 100 and 1000 µM of 4-hydroxybenzoic acid, 4-coumaric acid, and *trans*-ferulic acid for 28 days. Scale bars indicates 2 mm.

**
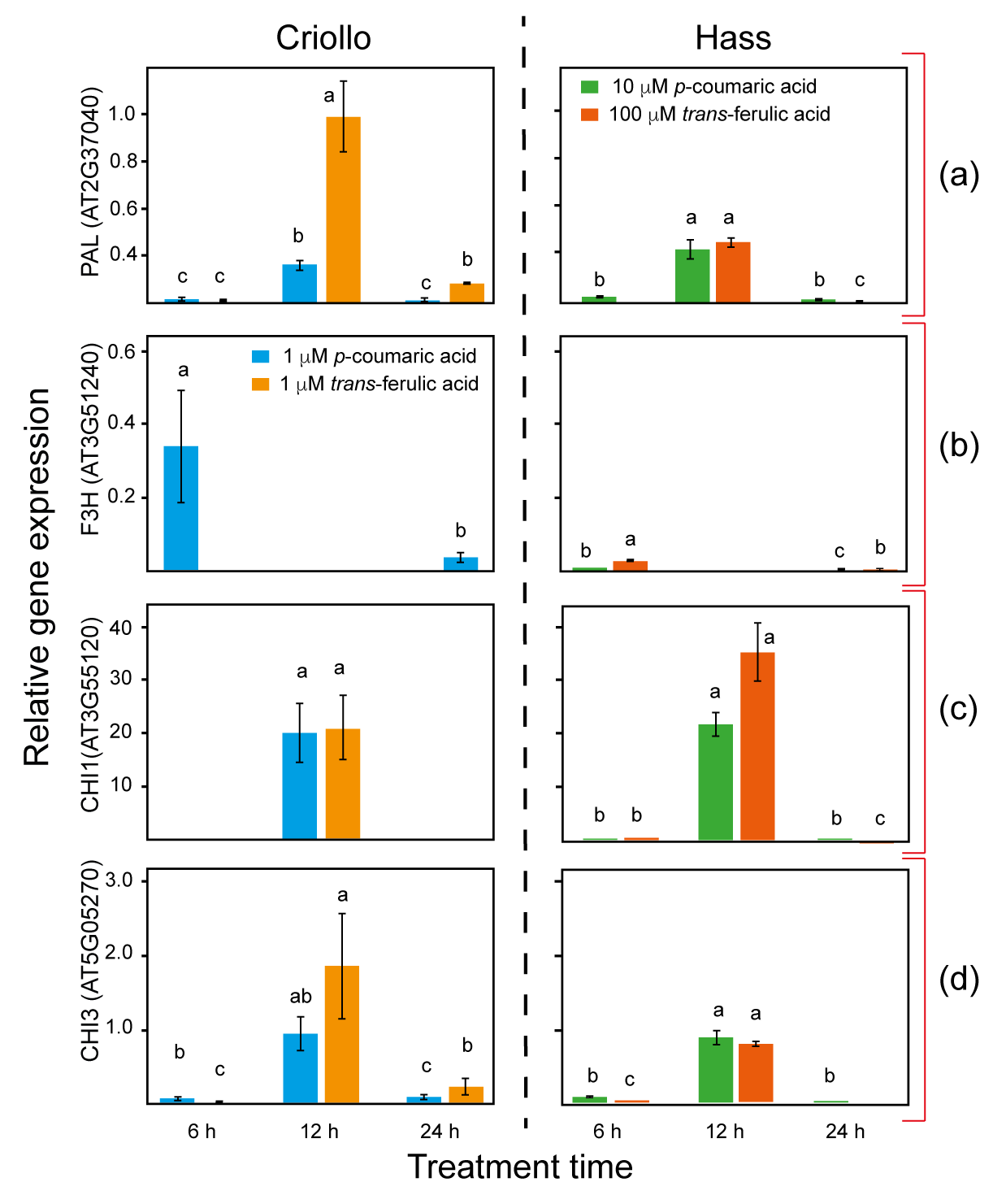
**

**Figure S6.** Quantitative real- time PCR validation of for genes involved in phenylpropanoids pathway in treatments with polyphenolic compounds. The gene expression was normalized using *Rubisco* as an internal control. Data are the means ± standard error of 3 biological samples. Different letters represent significant differences at *P* ≤ 0.05 by the Tukey´s test.

**Table S1**. Core proteome differentially identify in both Hass and Criollo EC and NEC (-), based on LIMMA (linear model for microarray data).

| **Unigene**  **(TAIR accession)** | | **Blast2GO**  **(UniProt Accessions)** | | **TAIR** **Annotation** | | **Peptides sequence** | **Criollo** | | **Hass** | |
| --- | --- | --- | --- | --- | --- | --- | --- | --- | --- | --- |
|  |  |  |  |  |  |  | **log2FC** | **-(P.Value)** | **log2FC** | **-(P.Value)** |
| *Stress related proteins* | | | | | | | | | | |
| FuEuUN011951 (AT2G22240) | B9T7K3 | | Myo-inositol-1-phosphate synthase 2 | | VLDIDLQK,  VGSFNGEEIYAPFK, VQQANYFGSLTQASSIR | | 3.290539279* | 7.36824E-05 | 0.695400997 | 0.082250521 |
| SusUN012375 (AT1G09750) | A0A061FET5 | | Spartyl protease AED3 | | ILFDLVNSR | | 2.924190987* | 0.000193653 | 1.398914565 | 0.009013499 |
| drymUN022975 (AT4G26010) | W9SDH9 | | Peroxidase 44 | | IGFYSSTCPQAESIIR, DAVSLAGGPNYLVPTGR, LFNFQGTGAPDSSMDSGLVAR, GFEIIDEAK | | 2.594073576* | 7.84557E-05 | 1.347564721 | 0.000968111 |
| drymUN024470 (AT3G21770/A0A1U7YQ23) | Q66RM0 | | Peroxidase 30 | | TFDLSYYNLLLK,  VEAIILDYVK,  GLFVSDAALITDSTSR,  FYEEFASSMEK,  LIEVQVK | | 2.506351252* | 0.000205446 | 0.893481469 | 0.011273502 |
| TolUN005584 (AT3G01420) | F6HUH2 | | α-dioxygenase 1 | | YNDPFNAIAGSEGTFFGR,  VHTIDWTVELLK,  FFTSDFNEETYTK,  NVPTGFYEIK,  LVTAAVIAK,  MHSLLPDTLLLR,  NHPDFSDEELYR | | 2.494441168* | 2.38725E-05 | 1.540910057* | 0.009704985 |
| PA10004118 (AT4G11650) | F6HUH2 | | Osmotin-like protein OSM34 | | VLNSYWINEDSTYK | | 2.349413799* | 1.60929E-05 | 1.748695652* | 0.001345936 |
| DryUN20261  (AT1G03220) | A0A0D2TUD9 | | Eukaryotic aspartyl protease family protein | | VAPVAPFGVCFSSK | | 2.270023311 | 4.46432E-05 | 0.595755091 | 0.336240299 |
| AnLuUN088718  (AT1G17860) | A0A061EZK2 | | Kunitz family trypsin and protease inhibitor protein | | WLGLSEVPFPVMFK, LQPNVEYYILPVIR, STVSNWFK | | 2.033235657 | 0.000786214 | 2.185966111 | 0.005828766 |
| hassUN035389 (AT1G14130) | F6I0A4 | | 2-oxoglutarate (2OG) and Fe(II)-dependent oxygenase superfamily protein | | LISVDPIPGSFLVNLGDVAK | | 1.667384804 | 2.87114E-05 | 0.889195842 | 0.001134069 |
| UN003689_Accession01  (AT5G42650) | A0A023UKW8 | | Allene oxide synthase | | VVDIVDTFR,  LQEQPAFDK | | 1.649682135 | 1.62223E-05 | 0.784063234 | 0.000924855 |
| drymUN034961 (AT2G47710) | A0A061DQR0 | | Adenine nucleotide alpha hydrolases-like superfamily protein | | SVTDVLVEVVEGDAR | | 1.595629595 | 0.000211615 | 1.467372366 | 0.000508715 |
| hassUN029102 (AT1G78830) | A0A061GEZ4 | | EP1-like glycoprotein 2 | | VAWQTGTANK,  CLLAPVLGTLTK,  FLWQSFDHPTDTLLVGQALR, VSNVDGSDGPYSFVLEAK,  LLPNGNLVLHDQQGR, LWEVTFTLFSR | | 1.537748379 | 0.001068688 | 1.411775239 | 0.003823875 |
| AnLuUN040913 (AT5G62390) | B9R9S1 | | BAG family molecular chaperone regulator 7 | | IGINGFGR | | 1.396191385 | 2.59825E-06 | 0.687533342 | 0.044986189 |
| DryUN10713 (AT1G71695) | F1DHX8 | | Peroxidase superfamily protein | | CGQIVSCSDIVALAAR, DATLNSLPGPSEK,  DIGQAAALLR,  MGQLSVLTGTK,  TPALIAALSR,  LYPTQDTTMDQTFSK | | 1.36146558 | 0.000485099 | 1.085234515 | 0.131171814 |
| UN024901_Accession01 (AT1G08080\|) | A0A061GYR3 | | Alpha carbonic anhydrase 7 | | IDEIFLR | | 1.258985348 | 0.000229047 | 0.989615141 | 0.182532075 |
| FuEuUN021440 (AT3G04880) | F6HTT7 | | DNA-damage-repair/toleration protein (DRT102) | | YFQDVVGK | | 1.174169886 | 0.00093746 | 1.311064937 | 2.25728E-05 |
| hassUN005288 (AT1G05180) | U5D564 | | NEDD8-activating enzyme E1 regulatory subunit AXR1 (Auxin-resistance protein AXR1) | | LIEDEFNSPNLSEFQK, NLVLGGIGSITVVDGSK, FVEEFPEDLIDR | | 1.15804974 | 0.00097934 | 0.651245958 | 0.012082315 |
| AnLuUN139617  (AT2G38870) | K0E192 | | Serine protease inhibitor, potato inhibitor I-type family protein | | SVWPELLGVAGEVAK | | 1.153638033 | 0.014119018 | 1.293767835 | 0.013130666 |
| TolUN042450 (AT1G16610) | U5U2Q2 | | Serine/arginine-rich splicing factor SR45 | | NYIEDLLK | | 1.104188516 | 0.002864532 | -0.819813566 | 0.000354451 |
| FuEuUN035399 (AT5G10230) | A0A061FYP5 | | Annexin D7 (AnnAt7) | | VIIEIACVSSPEELLAVK, MESELTGHLEK,  IISILAHR,  LAYEELYQQDLIR,  AFEGWGTNEK,  EAILVYTEVR,  SLPADSENEFFLALR,  LLVALVGTYR | | 1.080589407 | 0.001282564 | 1.134109032 | 0.004763349 |
| hassUN040691  (AT2G47730) | Q9M7I8 | | Glutathione *S*-transferase phi 8 | | EPFISLNPFGQVPALEDGDL, KEPFISLNPFGQVPALEDGDLK,  AITNYISHEYAGK, ELEFEFVPVNMATGDHK | | 1.052643925 | 0.000429588 | 1.833257987 | 3.29828E-05 |
| FuEuUN015523 (AT2G37220) | A0A0D2S4K1 | | RNA-binding protein CP29B, chloroplastic | | VYVGNLSWGVDDVALESLFSEQGK,  GFGFVTMSSLEEVK | | 0.943909573 | 0.011864939 | -1.124723411 | 0.001115153 |
| DryUN22839 (AT1G53680) | D2X9S1 | | Glutathione *S*-transferase TAU 28 | | FWADYIDK | | 0.940133136 | 0.008587219 | 1.864571362 | 0.006256028 |
| TolUN013883 (AT1G77120) | A0A0U2UPZ5 | | Alcohol dehydrogenase 1 | | AAVAWEAGKPLVIEEVEVAPPQVMEVR,  FITHEVPFSEINK, KPVQEVLAEMTGGGVDR, GTFFGNYKPR,  AFDYMLQGAGLR,  SDLPSVVEK,  FGVTEFVNPK, GSTVAVFGLGAVGLAAAEAR, FGVTEFVNPK,  FITHEVPFSEINK,  IIGVDLISSR,  INPDAPLDK,  KFGVTEFVNPK,  GVMIGDGQSR,  KFGVTEFVNPK,  AFDYMLQGAGLR | | 0.731670333 | 0.02267107 | 0.891633064 | 0.001374721 |
| DryUN46071  (AT5G59320) | G1DVA5 | | Lipid transfer protein 3 | | QASASISGIQPALAQGLPGK, | | 0.699184657 | 0.032076746 | 3.087698838 | 2.91335E-05 |
| hassUN038281 (AT2G21620) | 2049334 | | Adenine nucleotide α hydrolases-like superfamily protein | | LASLIPVVSEPELER, LAVEAFQVAMVK | | 0.690547368 | 0.00051094 | 0.726540154 | 0.024657587 |
| AnLuUN003219 (AT2G06050) | Q4ZJ73 | | 12-oxophytodienoate reductase 3 | | ISPAIDHLDAMDSDPLDLGLTVIK, LFISNPDLVLR | | 0.684146901 | 0.006946626 | 0.875369833 | 0.007643217 |
| AnLuUN068281 (AT5G58070) | Q38JB8 | | Temperature-induced lipocalin | | EDSTVNVLNETWTDGK,  WYEIASFPSR | | -0.583266368 | 0.004363703 | 1.803950911* | 0.000593354 |
| PA10027886 (AT2G13540) | A0A067JRI3 | | Nuclear cap-binding protein subunit 1 | | QVALIDEVSSFWK, LDAEVFVEDMHPLFR,  VMAAVEAYLR | | -0.585700157 | 0.007029532 | -0.621787779 | 0.001902847 |
| SusUN006923  (AT3G12580) | Q67BD1 | | Heat shock protein 70 | | VEIIANDQGNR, IINEPTAAAIAYGLDK, VEIIANDQGNR, SINPDEAVAYGAAVQAAILTGDTSEK | | -0.600541976 | 0.005723786 | 0.768465888 | 0.00197145 |
| PA10001224_Hass  (AT5G49910) | U5GKF8 | | Chloroplast heat shock protein 70-2 | | VVDWLAANFK, FDIDANGILSVTAIDK, SEVFSTAADGQTSVEINVLQGER, KQDITITGASTLPNDEVER, QAVVNPENTFFSVK,  FEELCSDLLDR,  IINEPTAASLAYGFEK, AVVTVPAYFNDSQR, MELSSLTQTNISLPFITATADGPK, VVDWLAANFKR, LSFKDLDEVILVGGSTR,  DEGIDLLK,  IAGLEVLR | | -0.6028504 | 0.00156056 | -0.635535303 | 0.017035668 |
| AnLuUN081428  (AT1G24020) | Q19VG6 | | MLP-like protein 423 | | IVSYSVIDGDIAAFYK, AKEEVPDPNIILEIAVK,  SIEIIEGDGK,  YTEGIPLVTFAK,  LEVEVEIK,  DLDAYLLK | | -0.603738486 | 0.133673412 | 2.198970442 | 0.000429236 |
| AnLuUN033157  (AT1G11910) | A0A061F5Z2 | | Aspartic proteinase A1 | | YGTGSVSGFFSQDNVQIGDLVVK, NAEEEEEEGGEIVFGGVDPK, FALTAEQYVLK, FTVIFDTGSSNLWIPSSK | | -0.609362201 | 0.011002715 | -1.158721098 | 4.50981E-05 |
| DryUN01129 (AT5G02500) | A0A067KKT3 | | Probable mediator of RNA polymerase II transcription subunit 37e | | VQQXLQDFFNGK, SINPDEAVAYGAAVQAAILSGEGNEK,  EQVFSXYSDNQPGVLIQVYEGER, VEIIANDQGNR, GEGPAIGIDLGTTYSCVGVWQHDR, TTPSYVAFTDSER,  FEELNMDLFR,  NALENYAYNMR,  FSDSSVQSDIK,  DAGVIAGLNVMR,  ELEGICNPIIAK, NQVAMNPINTVFDAK, NAVVTVPAYFNDSQR, IINEPTAAAIAYGLDK, MVNHFVQEFK,  ARFEELNMDLFR,  STIHDVVLVGGSTR,  FEELNMDLFR, | | -0.635977804 | 0.007530093 | -0.809366392 | 0.009278368 |
| SusUN047307 (AT1G27950) | B9GZQ5 | | Non-specific lipid transfer protein GPI-anchored 1 | | DTIEQQCSSALTK,  VMDCLQYSTGK, KDDPVCLCFFIQQTYK,  LLLLPTACK | | -0.638177919 | 0.009465934 | -0.957560614 | 0.076541163 |
| hassUN028055 (AT2G27260) | A0A068UEZ8 | | Late embryogenesis abundant (LEA) hydroxyproline-rich glycoprotein family | | LAALSSYVDDYDAR | | -0.658511322 | 0.002841727 | 0.795667578 | 0.013756492 |
| DryUN11369  (AT1G52820) | A0A067K7U9 | | 2-oxoglutarate (2OG) and Fe(II)-dependent oxygenase superfamily protein | | LFDLPLETK | | -0.711468135 | 0.049632631 | 1.206257782 | 0.000254071 |
| DryUN27351 (AT5G56950) | M5WU00 | | Nucleosome assembly protein 1;3 | | LDFFFDPNPYFK,  TYHMIDDDEPILEK,  AIGTEIEWFPGK | | -0.922806041 | 0.001150914 | -0.675830437 | 0.011610542 |
| hassUN011252 (AT5G61820) | A0A061FZN9 | | Stress up-regulated Nod 19 protein | | TMVYPEPGFELER,  VEPLVNMGQISR | | -0.934291434 | 0.002170785 | 0.661182145 | 0.002024334 |
| AnLuUN039666 (AT1G77510) | B9RJ25 | | Protein disulfide isomerase-like 1-2 | | FVEEASVPVITIFNK, GYPTLYFSSASGK,  SISFLLGDLK | | -1.000810635 | 0.001748771 | -0.955385197 | 0.00158404 |
| drymUN024472  (AT1G01820) | W9QYS0 | | Peroxin 11c | | LFDLPLETK | | -1.198312807 | 0.00280792 | 1.097091074 | 0.005295673 |
| AnLuUN072812 (AT3G09270) | A0A061ESJ9 | | Glutathione *S*-transferase TAU 8 | | HMQEAMESLK, GIEYEYIEEDLSNKSPLLLK, GIEYEYIEEDLSNK,  LFGAWGSPFSR,  TLETALQGK,  LKGIEYEYIEEDLSNK | | -1.353341231 | 0.00015428 | 1.405916179 | 0.001495463 |
| PA10002345_Hass  (AT1G08830) | D5MQD6 | | Copper/zinc superoxide dismutase 1 | | VACGVIGLQA | | -2.029421383 | 1.40123E-06 | 0.710818617 | 0.118236315 |
| SusUN018282 (AT3G12490) | M5X8D6 | | Cysteine proteinase inhibitor 6 | | SNSLTPYELLEILHAK, NVEGTFLLNQMQQEHN,  AEVIEEFAK,  QNALLEFAR, AEEQVVAGTLHHLTVEVIDAGK, NVEGTFLLNQMQQEHN | | -2.562699628* | 1.69917E-08 | 0.66328155 | 0.00399423 |
| *Proteins associated with phenylpropanoid metabolism* | | | | | | | | | | |
| drymUN023723 (AT5G05340) | Q7XYR7 | | Peroxidase 52 | | IYTETNIDSSFAR, AYYNSPSSFNADFAAAMIK, VCPGVVSCADILAIASR, GLLHSDQQLFNGGSTDSQV,  LISSFSSK | | 3.474455378 | 5.32794E-06 | 1.503113323 | 0.01716724 |
| TolUN022975 (CCoAOMT1, AT4G34050) | Q6J524 | | Caffeoyl-CoA *O*-methyltransferase 1 | | EGPAMPVLDQMIEDK,  DFVLELNK, VGGVIGYDNTLWNGSVVAPPDAPLR,  SLLQSDQLYQYILETSVYPR, SLLQSDQLYQYILETSVYPR | | 3.172579662* | 0.000169803 | 2.266153862* | 0.000171884 |
| AnLuUN030269 (4CL1, AT1G51680) | I6PD12 | | 4-coumarate: CoA ligase 1 | | VTIAPFVPPIVLAIAK | | 2.824627814* | 0.000371134 | 1.804163738* | 0.000380025 |
| PA10003761_Hass (AT5G05270) | A0A061FTJ1 | | Chalcone-flavanone isomerase family protein | | AVSPTTVASLASGLGALLSK, DDLFYDSLISAPVEK | | 2.419980785* | 1.82333E-07 | 0.807228289 | 0.08643062 |
| hassUN012729 (AT3G51240) | A0A061DFA7 | | Naringenin,2-oxoglutarate 3-dioxygenase | | EIVTFFSYPIR,  EFFTLPAEEK | | 2.377322757* | 1.34942E-07 | 0.59600262 | 0.046529849 |
| FuEuUN005076 (AT2G37040) | I6P9B7 | | Phenylalanine ammonia-lyase 1 | | TSPQWLGPQIEVIR, LAGIPSGFFELQPK | | 2.229904645* | 0.000201962 | 0.701114465 | 0.07386675 |
| DryUN12037 (LDOX, AT4G22880) | X4RA14 | | Leucoanthocyanidin dioxygenase | | EELTSIVDVFEEEK,  AFFDLPIEQK,  EFGGMEEFLLQMK, VEQLTGLQAIPVEYVRPK | | 1.802909261 | 2.00643E-05 | 0.70729071 | 0.0027462 |
| DryUN12331 (OMT1, AT5G54160) | Q9SYR9 | | Flavone 3'-O-methyltransferase 1 | | TVVDVGGGTGAVLNMIISK, AAIELDVLEIIAK,  EIASQLPTK | | 1.571832444 | 0.000681721 | 1.421719821 | 0.000619628 |
| hassUN036909 (AT3G55120) | A0A059VKQ3 | | Chalcone--flavonone isomerase 1 | | FMQVTMILPLTGQQYSEK, GQELTDAVDFFR, AVGLYTEAEAIAVEK, DSSIPEQAAGVIENK | | 1.13183479 | 1.12084E-05 | 0.635060718 | 0.005365982 |
| PA10021556 (CYP7135, AT3G26310) | W9RB74 | | Cytochrome P450, family 71, subfamily B, polypeptide 35 | | AQQELETMVGK | | 0.852087109 | 0.006934268 | 2.050937062* | 0.000872289 |
| drymUN010612 (AT5G49690) | A0A061DUY2 | | UDP-Glycosyltransferase superfamily protein | | VTLLPHEANGLFPAFYLPNGSGLSDIQR,  YFGSFVDFLK,  SVVFVGFGSECNLSR, TEEAGGGEWVEIFR, VTLLPHEANGLFPAFYLPNGSGLSDIQR,  ISFLSTPK | | 0.738210509 | 0.001557133 | 1.373311637 | 0.000256938 |
| AnLuUN028663 (ALH2C4,  AT3G24503) | M5W1P1 | | Aldehyde dehydrogenase family 2 member C4 | | FANLIDENLEELAALDSLDGGK, LFINGEFVDAISGK | | -0.827747864 | 0.002624464 | 0.797485105 | 0.05213778 |
| hassUN023265 (CCoAOMT, AT4G26220) | F6H5G8 | | Probable caffeoyl-CoA *O*-methyltransferase | | IEFIESPALPVLDK, ITAIDTDRDAYNIGLPIIR | | -1.089088603 | 9.67806E-05 | 1.000363238 | 0.000726121 |
| hassUN036924  (AT4G15550) | A0A125QVX4 | | UDP-glycosyltransferase 75D1 (quercetin 3-O-glucosyltransferase activity) | | DAPDFLALFSQAGAR,  VLVNSFDELER | | -1.323528514 | 1.56478E-05 | 0.736200445 | 0.014707714 |
| drymUN019539 (CAD9, AT4G39330) | W9R066 | | Cinnamyl alcohol dehydrogenase 9 | | VTVISTSPSK, FPDNLPLDAGAPLLCAGITVYSPLK, FVIDIANTLK, LVMVGAPDRPLELPVFPLIMGR, NEWGFSFYPAVPGHEIVGVVTEVGK,  IVAGSCIGGMK,  ETQEMIDFAAK, HNITADIEVIPMDYVNTAMER, NEWGFSFYPAVPGHEIVGVVTEVGK,  LGADSFLVSR, FPDNLPLDAGAPLLCAGITVYSPLK, SGVLSPFK | | -1.89423896 | 5.62577E-05 | -0.97867146 | 0.000734296 |
| *Carbohydrate metabolic process* | | | | | | | | | | |
| DryUN33954 (AT3G57270) ^a^ | Q9M563 | | Probable glucan endo-1,3-β-glucosidase BG1 | | | YINVGNELIPGGLAQFILPAMR, NIYNAIGSAGLRDK, NIYNAIGSAGLR,  NFGLFYPSK, FKYINVGNELIPGGLAQFILPAMR, VSTAIDMGVLGNSFPPSXGSYK | 2.847479786* | 1.42836E-06 | 2.063149912* | 0.002296542 |
| PA10046698_Hass (AT4G16260) ^a^ | A0A0F6TMS2 | | Probable glucan endo-1,3-β-glucosidase | | | APLLANVYTYFSYAGNPK, DISLQYALFTSPSVVVQDGQLK, YQNLFDAMLDALYSAVER, VSTAIDMGVLGNSFPPSQGSFK, NIYNAIGSAGLR,  QPVYSINFSPR,  LYDPNQAALEALK, GSNIEVLLGVPNQNLQEMA, NIYNAIGSAGLRDK,  NFGLFYPSK,  QYLEPIVRFLASTR, VSTAIDMGVLGNSFPPSQGSFK, EVVALYK | 2.647259543* | 2.10073E-06 | 1.238426319 | 0.017497683 |
| TolUN068057 (AT3G54420) ^a^ | F8V482 | | Homolog of carrot EP3-3 chitinase | | | TAFWFWMNNCHDAIIQDR, GPLQLTWNYNYGK, AGQALGFDGLNNPDIVSR | 2.488579836* | 7.98159E-06 | 0.934141047 | 0.067387252 |
| AnLuUN028051  (AT5G01320) | Q1I1D9 | | Thiamine pyrophosphate dependent pyruvate decarboxylase family protein | | | LSNQIGLEAAVEATAEFLNK, TEEELVEAIATVMGAQK, DPVPFFLAPR, VRTEEELVEAIATVMGAQK, LSNQIGLEAAVEATAEFLNK, IYVPPGVPLK | 1.497860477 | 2.88006E-05 | 0.773650013 | 0.03310151 |
| DryUN09346  (AT2G36460) ^a^ | Q9M4M9 | | Aldolase superfamily protein | | | LASVNVENVESNRR, TVPPAVPAIMFLSGGQSEEEATLNLNAMNK,  KVTPEVVAEYTVR, ANSEATLGIYKGDAAK, LASVNVENVESNR,  SSSGKPFVEILK,  EGGVLPGIK,  ANSEATLGIYK,  ANSEATLGIYK, GILAADESTGTVGK, YTDELIANAAYIGTPGK, IGPTEPSQLAINESANGLAR, VTPEVVAEYTVR, CAIICQENGLVPIIEAEILVDGPHSIEK, VDTGYVDXAGINGEIATQGLDGLAQR,  TWVGKEENILK, KVTPEVVAEYTVR, TVPPAVPAIMFLSGGQSEEEATLNLNAMNK | 1.443905718 | 0.000314424 | 0.935586244 | 0.031113015 |
| AnLuUN035495 (AT3G18080) | T2DPW3 | | β-glucosidase 44 | | | AFADFADFCFK, GFIFGTATSAYQVEGMAR, VVAALGYDNGIFAPGR,  SYLLQLEK,  IFPYGVGK,  LIDYLLER | 1.373430435 | 3.2468E-06 | 0.997862507 | 0.001815808 |
| PA10017334_Hass  (AT1G55850) | L0AUS3 | | Cellulose synthase like E1 | | | EGYLPLFETK | 1.233025344 | 0.000619545 | 1.473749651 | 0.000291377 |
| DryUN11228 (AT3G12500) ^a^ | P93680 | | Basic chitinase | | | GFYTYNAFIAAANSFNGFASVGDTATR,  GFYTYNAFIAAANSFNGFASVGDTATR,  AIGYDLINNPDAVATDPVISFK,  LPGYGVITNIINGGLECGK,  AAGRLPGYGVITNIINGGLECGK,  YCDLLGVSYGSNLDCYNQR, GPIQISYNYNYGPAGR | 1.049867822 | 0.000665962 | 1.070918718 | 0.001764195 |
| AnLuUN103515 (AT3G46940) | A0A0B0PFT1 | | Deoxyuridine 5'-triphosphate nucleotidohydrolase | | | ALVPTDLSIGIPEGTYAR, IVTPEVLLVDDLDSTVR,  IAQLILEK | 0.987574956 | 0.026054378 | -1.884012702 | 4.41752E-05 |
| DryUN00817 (AT4G02280) ^a^ | A0A140GPJ4 | | Sucrose synthase 3 | | | LMTLAGVYGFWK,  NLTGLVEWFGK,  VVHGIDVFDPK,  NITGLVEWFGK,  LTSLLPSIEK,  NELISLLLR | 0.917796332 | 6.72999E-05 | 1.491870027 | 6.04904E-05 |
| hassUN023342 (AT5G08380) | H6WD96 | | Alpha-galactosidase 1 | | | TFAEWGIDYLK, LPITALWEDIELPVDTVVK | 0.878929146 | 0.004445969 | -0.668284541 | 0.002648862 |
| UN010433_Accession01 (AT2G45220) ^a^ | B9SP63 | | Probable pectinesterase/pectinesterase inhibitor 17 | | | AGLYSENVEITR,  SATFAISGDGFIAR, NLMFIGDGIDSTIVTGSK, GYQDTLYVYSQR | 0.652011389 | 0.002244118 | 0.952982643 | 0.015470095 |
| TolUN010227  (AT5G57655) | A0A061FT64 | | Xylose isomerase family protein | | | YQSFDTEIGALIEAGK,  NGGLAPGGFNFDAK,  EGYQSLLNTDMK,  ADFELLEK | 0.590649597 | 0.023805538 | 0.834877895 | 0.000952982 |
| drymUN010967 (AT1G66430) | 2028987 | | Probable fructokinase-6, chloroplasti | | | EGILSIWDTADIIK,  ISEEEISFLTK,  VGADEFGYMLADILK | -0.651845524 | 0.000994685 | 0.637593225 | 0.004963233 |
| drymUN014922  (AT5G24318) | A0A061GVZ6 | | *O*-Glycosyl hydrolases family 17 protein | | | LPAAQSWVSSNISPFLPATK | -0.958181778 | 0.00174018 | -0.983926656 | 0.000161654 |
| AnLuUN047960 (AT2G43590) ^a^ | D7P708 | | Endochitinase | | | EVAAFLANVMHETGGLCYVR, GPLQLSWNYNYGAAGK, LTNGGGFASTIR,  FLANLPASCPGK,  GFYTYNGIIR, GFGTTGSVTDR | -1.086475051 | 0.002747237 | 0.743670504 | 0.154240921 |
| SusUN005127 (AT4G23820) | A0A097PQY7 | | Pectin lyase-like superfamily protein | | | ADVVSIVDFGGVGDGR | -1.525336643 | 7.22742E-05 | -0.825478151 | 0.018552736 |
| *Miscellaneous* | | | | | | | | | | |
| DryUN01514 (AT4G23850) | A0A0M4FYS7 | | Long chain acyl-CoA synthetase 4 | | | LILSGAAPLAAHVEAFLR,  EYILGELIK | 1.179337883 | 1.01863E-05 | 0.670931875 | 0.001438904 |
| DryUN40895 (AT1G65820) | J3LS26 | | Microsomal glutathione s-transferase, putative | | | VFYPTLYAIESENK | -0.593274666 | 0.005270453 | 0.864779593 | 0.000272768 |
| hassUN025208 (AT3G51730) | A0A061EI06 | | Saposin B domain-containing protein | | | LEIIELLLK | -0.666270254 | 0.000833194 | 0.889691065 | 0.001943285 |
| drymUN012527 (AT5G59420) | D7SKD8 | | OSBP (oxysterol binding protein)-related protein 3C | | | FLGNSVDVYPVGR, ASIDAADIINEVDVK | -1.770068529 | 1.04279E-05 | -0.891565638 | 0.000148441 |
| SusUN079154 (AT3G43720) | I1NE15 | | Non-specific lipid transfer protein GPI-anchored 2 | | | SNSFGIDLDMAK | -2.107121999* | 7.81137E-07 | -0.905507051 | 0.004148941 |
| drymUN021084 (AT1G29660) | A0A061GDB6 | | GDSL esterase/lipase | | | QYTPEQFADLLIQQYSEQLR, LATLVEDLNDSLDSAK, GEEILTGVNFASAAAGIR, TTVDVIAELLGFDNYIPPYTTAR | -2.412595442* | 0.000221457 | 0.676340724 | 0.16997459 |
| UN29726_Drymifolia  (AT5G39160) | U5DBD4 | | RmlC-like cupins superfamily protein | | | DVTAQDFFYEGLDKPGDTGNK | 4.350920316 | 8.52677E-07 | 0.675402584 | 0.238029271 |
| drymUN018899 (AT2G45600) | A0A061DTS9 | | Probable carboxylesterase 8 | | | IDGLILNEPAFGGVER, FKIDGLILNEPAFGGVER, LPAAYDDAIDAIR | 3.524330745* | 1.98238E-06 | 0.951081763 | 0.021191772 |
| drymUN035223  (AT1G19130) | W1NV20 | | RmlC-like jelly roll fold | | | DIDAYSSDGSSLVK,  AHPEGGFYSETFR | 2.845462907 | 4.29406E-07 | 0.628437173 | 0.003668902 |
| PA10128055 (AT3G26770) | D3JX99 | | Alcohol dehydrogenase-like protein | | | VLSVNLVGGLLGTK | 2.730938374* | 7.27475E-05 | 0.937636706 | 0.003698718 |
| DryUN06456 (AT5G54080) | A0A061F763 | | Homogentisate 1,2-dioxygenase | | | DFLAPTAWFEDDFLPGYTIVQK, LQVSPGEIVVLPQGFR | 1.812563064 | 1.12476E-06 | 0.687577636 | 0.006300118 |
| DryUN31781 (AT5G41850) | A0A072U5T9 | | Alpha/beta-Hydrolases superfamily protein | | | AIAEFIFK | 1.811124846 | 8.9959E-06 | -0.708456739 | 0.009319866 |
| AnLuUN075450 (AT4G35785) | A0A0K9NQX3 | | RNA-binding (RRM/RBD/RNP motifs) family protein | | | GFAFVTMDSLEDADR | 1.554727642 | 5.99108E-05 | -0.917643195 | 0.004780987 |
| FuEuUN005657 (AT5G48960) | W1NWK4 | | HAD-superfamily hydrolase, subfamily IG, 5'-nucleotidase | | | VVVALPNELLSAVAR | 1.544472223 | 7.8325E-05 | 0.718999495 | 0.002044971 |
| hassUN006884(AT3G57150) | F6HLD3 | | H/ACA ribonucleoprotein complex subunit 4 | | | VVMPLEVVLTSYK, DYLIKPQSFTPSLDTSQWPILLK, ALETLTGAVFQRPPLISAVK, LVVEGLLDK | 1.518694588 | 9.49676E-06 | -0.880592577 | 0.001583399 |
| UN005206_Accession02 (AT5G25880) | A0A061FML7 | | NADP-malic enzyme 3 | | | LLIDNVEELLPIVYTPTVGEACQK | 1.186092894 | 0.001894392 | 1.609634532* | 0.003059483 |
| drymUN026870 (AT3G09140) | W9RHQ6 | | Protein of unknown function (DUF674) | | | NVNIGAAEVLHLLK,  SVFTDVFYPK, EFGGSFIAGPAMFLVTDELNVK | 0.778159927 | 0.157355412 | -1.606431174* | 3.51278E-05 |
| AnLuUN056387 (AT1G32210) | Q84L93 | | Dolichyl-diphosphooligosaccharide--protein glycosyltransferase subunit (Defender against death DAD family protein | | | DAQALIQSLR | 0.675295322 | 0.012978098 | 2.202137762* | 0.008214478 |
| AnLuUN041788 (AT5G28840) | A0A061F2D5 | | GDP-D-mannose 3',5'-epimerase | | | SFTFIDECVEGVLR, AITSTDKFEMWGDGLQTR, ISITGAGGFIASHIAR, VVGTQAPVQLGSLR, SQGVDLSVYGSSK, ESDAWPAEPQDAYGLEK, QLETNVSLK,  ITYFWIK | 0.655472117 | 0.006329978 | 0.981106273 | 0.000833892 |
| hassUN002653 (AT5G15270) | A0A061EMF5 | | RNA-binding KH domain-containing protein | | | TNAFDDSGDYISPAQDALFR, LAADEAPGEEDEEDVEAHQITVR, EFFEDPISPSIDAAVR, LLVPSDQIGCIIGK | 0.625213748 | 0.000860479 | -0.869015243 | 0.005816016 |
| DryUN02825 (AT5G01460) | A0A061ES69 | | LMBR1-like membrane protein | | | ELLLLEDDVK | 0.614162369 | 0.005283024 | -0.628009319 | 0.012652455 |
| drymUN016592 (AT2G27920) | D7SKD8 | | Serine carboxypeptidase-like 51 | | | LGTITPMDQVLWR | -0.622308302 | 0.004002629 | -1.275155964 | 2.06903E-05 |
| PA10000713 (AT5G61310) | M0RU02 | | Probable cytochrome c oxidase subunit 5C-3 | | | AFYDMLEK | -0.627601969 | 0.022977751 | -0.649258085 | 0.003503409 |
| DryUN16854 (AT5G24650) | A0A061G2Z0 | | Mitochondrial import inner membrane translocase subunit Tim17/Tim22/Tim23 family protein | | | GLLTDSTLPLLTDSALR, SMLTNLGLQNYEK,  LLILDHIQR | -0.645511674 | 0.000439599 | -0.717287594 | 0.004616952 |
| drymUN019750 (AT5G17710) | A0A061F7B9 | | Co-chaperone GrpE family protein | | | EESTEFEEGIIIQEFR, LSLVTNVQGEVVENLLPVLDNFER | -0.657328909 | 0.008261527 | 1.323034247 | 7.01548E-05 |
| DryUN02370 (AT1G71770) | W9QI86 | | Polyadenylate-binding protein 5 | | | VMLGEQLFPLVER, ELFTEFGTITSCK,  LQGANLYLK, NLSETTTDEDLKNIFGNYGPITSAVIMR | -0.674092343 | 0.000265181 | 0.672003066 | 0.004148336 |
| hassUN028475 (AT1G24450) | D7SHV0 | | Protein NUCLEAR FUSION DEFECTIVE 2 | | | QIGYQFQTVDLLR, TDVSAPVVLCGAFR,  ILSYLGLR | -0.838722019 | 0.000331571 | 0.931369239 | 0.002478832 |
| FuEuUN012428 (AT2G45470) | B9RDF1 | | Fasciclin-like arabinogalactan protein 8 | | | NALALLTLLDYYDAQK, TATTPISTLASSGAGK, TFASLILSSGVLK,  GLTVFAPSDEAFK | -0.926353711 | 0.001072516 | 1.551426431 | 0.004674104 |
| FuEuUN018457 (HDA3, AT3G44750) | M1AFH0 | | Histone deacetylase HDT3 | | | SILGSVGAEVEEDRIDFLLTELK, GKDITELIASGR,  DITELIASGR | -0.933295677 | 0.002243162 | -1.261205684 | 0.000566944 |
| hassUN040266 (AT5G10260) | W9QNC0 | | Ras-related protein RABH1e | | | DSSVAVIVYDVANR, GSDVIIVLVGNK,  QSFLNTSK,  LVFLGDQSVGK | -1.347245661 | 2.13701E-05 | 0.786424653 | 0.007886022 |
| AnLuUN030696 (AT1G76160) | B9SR02 | | SKU5 similar 5 | | | VSGPPPGGPTIQVDWSLNQAR, YGVNSVSFIPADTPLK, TIILANSAGQVNGK,  AAGAFGGIK,  FPDGILINGR, YGVNSVSFIPADTPLK, QYLGQQFYLR, DQIGSFFYFPSLAFHK, IPVPFPDPAGDFTVLIGDWYTK | -1.884344576 | 3.10151E-06 | -0.725174081 | 0.079356914 |
| FuEuUN017407 (AT1G26160) | W1PF78 | | Metal-dependent phosphohydrolase | | | MALMALIAGDLQGVNR | -2.288135173* | 6.10012E-06 | 0.607055451 | 0.023368537 |
| PA10024027 (AT1G44130) | Q2QWY7 | | Eukaryotic aspartyl protease family protein | | | AQLEIAPEGYLIITR | -2.76823551* | 5.00873E-07 | -0.805713798 | 0.018905408 |
| PA10016280_Hass  (AT3G48530) | W9RMC0 | | SNF1-related protein kinase regulatory subunit gamma 1 | | | DVQFLLSAPEIYK | 1.78067389 | 1.11137E-06 | 0.959590672 | 6.11731E-05 |
| DryUN08470  (AT4G35630) | F6H8F3 | | Phosphoserine aminotransferase | | | LVAFMNDFQAK,  FGIIYAGAQK,  SLMNVPFTLEK,  AQSELFNWR | 1.13789893 | 5.58809E-06 | 0.745319185 | 0.001109432 |
| DryUN07928  (AT4G13010) | A0A0D2N7W9 | | Oxidoreductase, zinc-binding dehydrogenase family protein | | | VVSILGATTGGGLAEYAVAQAK, NLGADEVLDYR | -0.652193476 | 0.012889691 | 0.838387528 | 0.000749587 |
| DryUN24255  (AT5G42190) | A0A0S2C4U1 | | E3 ubiquitin ligase SCF complex subunit SKP1/ASK1 family protein | | | SLLDLTCQTVADMIK, | -0.72907012 | 0.008497162 | -0.601529136 | 0.140442986 |
| PA10017130_Hass  (AT3G23750) | A5AXB9 | | Leucine-rich repeat protein kinase family protein | | | LAGTFGYLAPEYAATGR | -0.81569 | 0.002373 | -0.65884 | 0.008217 |
| PA10029507_Hass (AT5G53220) | A0A059DIP2 | | Unknown protein | | | ILQLEEDNSNLR,  LEVEVEQEAQEK | -1.005487202 | 6.88168E-05 | -0.583240924 | 0.024223271 |
| hassUN009836 (AT1G60500) | A0A061EXZ4 | | Dynamin related protein 4C | | | SSVLESLAGISLPR, EGIQLPTIVVVGDQSSGK | -1.086896478 | 0.000372294 | -0.932954903 | 0.015722571 |
| AnLuUN025755 (AT1G43710) | U5D3K0 | | Pyridoxal phosphate (PLP)-dependent transferases superfamily protein | | | GAVDDLDLVIK,  IDDFMSELLHK | -1.287355742 | 2.00364E-05 | -1.003623432 | 0.000255777 |
| PA10019795 (AT3G10150) | A0A059DIP2 | | Purple acid phosphatase 16 | | | IIDITEQPFSIK, SQEINPGSTVPELVFWHIPSK, LELMEGEIER | -1.407246092 | 2.78699E-05 | -1.414808559 | 0.001154493 |
| FuEuUN023689 (AT3G02870) | A0A067K9K2 | | Inositol monophosphatase family protein | | | GENDSLAEFLAVAVDAAK | -1.717889087 | 0.000503819 | -1.035742362 | 0.000253216 |
| TolUN048298 (AT2G44310) | A9PCU6 | | Calcium-binding EF-hand family protein | | | FAALDLNSDGVLSR | -1.956622671 | 3.30251E-07 | 1.331431176 | 0.000204297 |
| drymUN025670 (AT5G65205) | F6H4N8 | | NAD(P)-binding Rossmann-fold superfamily protein | | | TFGIDVITVVPGAIK | -2.092634636 | 1.94318E-05 | -1.29180544 | 4.06042E-05 |
| hassUN021465 (AT1G71750) | W9RXW5 | | Hypoxanthine-guanine phosphoribosyltransferase | | | LPPIDEQIISMK | -4.903077348* | 3.6069E-10 | -0.605726184 | 0.067124911 |

^a^Proteins associated with carbohydrate metabolic process and stress related response. *Differentially expressed proteins with significant accumulation base on linear model for microarray data (LIMMA). For detail information reviewed Supplementary Table S1

**Table S2.** Statistical analysis of values associated with the endogenous content of polyphenolics (μg g-1 fresh weight) in embryogenic Criollo and Hass cultures treated with different concentrations of *p*-coumaric acid *and trans*-ferulic acid during six h, twelve h, 14 and 28 days.

| Cultivar | Treatment | Time (h) | 4-hydroxy benzoic acid | Cafeic acid | Vanillin | *p*-cumaric acid | Quercetin 3,4´-di-*O*-glucoside | Ferulic acid | Sinapic acid | Naringin | Trans-cinnamic acid | Naringenin |
| --- | --- | --- | --- | --- | --- | --- | --- | --- | --- | --- | --- | --- |
| **Criollo** | Control | 0 | 0.11 ±0 | 0 | 0.11±0.01 | 0.42±0.01 | 0.02±0 | 0.23±0 | 0 | 0.07±0.01 | 0.02±0.01 | 0.02±0 |
|  | *p*-CA1 |  | 0.11 ±0 | 0 | ***0.11±0.01*** | ***0.42±0.01*** | ***0.02±0*** | ***0.23±0*** | 0 | 0.07±0.01 | 0.02±0.01 | 0.02±0 |
|  | *t*-FA1 |  | 0.11 ±0 | 0 | ***0.11±0.01*** | ***0.42±0.01*** | 0.02±0 | 0.23±0 | 0 | 0.07±0.01 | 0.02±0.01 | 0.02±0 |
| **Hass** | Control |  | 0.21±0.01 | 0 | 0.03±0.03 | 0.66±0.02 | 0.01±0.01 | 0.09±0.01 | 0 | 0 | 0.04±0 | 0 |
|  | *p*-CA1 |  | 0.21±0.01 | 0 | 0.03±0.03 | 0.66±0.02 | 0.01±0.01 | 0.09±0.01 | 0 | 0 | 0.04±0 | 0 |
|  | *p*-CA10 |  | 0.21±0.01 | 0 | 0.03±0.03 | 0.66±0.02 | 0.01±0.01 | 0.09±0.01 | 0 | 0 | 0.04±0 | 0 |
|  | *t*-FA1 |  | 0.21±0.01 | 0 | 0.03±0.03 | 0.66±0.02 | 0.01±0.01 | 0.09±0.01 | 0 | 0 | 0.04±0 | 0 |
|  | *t*-FA100 |  | 0.21±0.01 | 0 | 0.03±0.03 | 0.66±0.02 | 0.01±0.01 | 0.09±0.01 | 0 | 0 | 0.04±0 | 0 |
| **Criollo** | Control | 6 | 0.58±0.01^***^ | 0^***^ | 0.22±0^***^ | 0.85±0.01^***^ | 1.66±0.07^***^ | 1.12±0.01^***^ | 0.7±0.01^***^ | 0.34±0.01^***^ | 0.06±0^**^ | 0.12±0.01^***^ |
|  | *p*-CA1 |  | 0.64±0.05^***^ | 0^***^ | ***0.06±0.01^**^*** | **0.29±0.01^**^** | ***0^*^*** | **0.12±0.01**^**^ | 0^**^ | 0^**^ | 0.4±0^***^ | 0^*^ |
|  | *t*-FA1 |  | 0.38±0.01^**^ | 0^***^ | ***0.05±0^**^*** | **0.18±0.01**^**^ | 0.03±0.04^**^ | 0.28±0^**^ | 0^**^ | 0^**^ | 0.11±0.01^***^ | 0.05±0^**^ |
| **Hass** | Control |  | 0.29±0.01^*^ | 0^***^ | 0.06±0^*^ | 0.62±0.01^*^ | 0.33±0.05^*^ | 1±0.01^**^ | 0.86±0.02^***^ | 0.06±0.01^*^ | 0.03±0^*^ | 0.02±0^*^ |
|  | *p*-CA1 |  | 0.52±0^**^ | 0^***^ | 0.1±0.01^*^ | 0.49±0.01^*^ | 0.45±0.02^*^ | 0.29±0.01^**^ | 0^*^ | 0.1±0.01^**^ | 0.65±0.01^**^ | 0.14±0^**^ |
|  | *p*-CA10 |  | 0.25±0^*^ | 0^***^ | 0.05±0^*^ | 0.7±0^**^ | 0.02±0.03^**^ | 0.13±0.01^*^ | 0^*^ | 0^*^ | 0.29±0^*^ | 0.01±0^*^ |
|  | *t*-FA1 |  | 0.55±0.01^***^ | 0^***^ | 0.14^**^ | 0.35±0.01^*^ | 0.35±0.02^*^ | 0.5±0.01^*^ | 0.13±0.01^*^ | 0.1±0.02^**^ | 0.46±0.01^*^ | 0.15±0^***^ |
|  | *t*-FA100 |  | 0.32±0.01^*^ | 0^***^ | 0.24±0^***^ | 1.39±0.01^***^ | 0.62±0.02^***^ | 10.6±0.22^***^ | 0.64±0.02^**^ | 0.18±0.02^***^ | 0.98±0.02^***^ | 0.11±0^*^ |
| **Criollo** | Control | 12 | 0.21±0^**^ | 0^***^ | 0.15±0.01^***^ | 0.61±0.01^***^ | 0.62±0.04^***^ | 0.52±0.01^***^ | 0.72±0.02^***^ | 0.05±0.01^***^ | 0.02±0^**^ | 0.05±0.08^***^ |
|  | *p*-CA1 |  | 0.25±0^***^ | 0^***^ | 0.06±0.01^**^ | 0.46±0.01^**^ | 0^**^ | 0.26±0.01^**^ | 0^**^ | 0^***^ | 0.15±0.01^***^ | 0.04±0^***^ |
|  | *t*-FA1 |  | 0.18±0^*^ | 0^***^ | 0.04±0.01^*^ | 0.18±0.01^*^ | 0^**^ | 0.12±0.01^*^ | 0^**^ | 0^***^ | 0.03±0.01^**^ | 0^***^ |
| **Hass** | Control |  | 0.6±0.01^***^ | 0* | 0.11±0.01^*^ | 0.94±0.02^***^ | 0.18±0.02^***^ | 0.86±0.07^***^ | 0.42±0.01^***^ | 0.21±0.01^***^ | 0.12±0.01^**^ | 0.2±0^***^ |
|  | *p*-CA1 |  | 0.44±0.01^*^ | 0.9±0.01^**^ | 0.08±0^*^ | 0.28±0.01^*^ | 0.06±0.02^**^ | 0.21±0.01^*^ | 0^*^ | 0.05±0.01^*^ | 0.08±0.01^*^ | 0.04±0.01^*^ |
|  | *p*-CA10 |  | 0.2±0^*^ | 0^*^ | 0.03±0^*^ | 0.24±0^*^ | 0.04±0^**^ | 0.13±0^*^ | 0^*^ | 0^*^ | 0.08±0.01* | 0.03±0^*^ |
|  | *t*-FA1 |  | 0.57±0.01^**^ | 0.11±0.02^**^ | 0.09±0.01^**^ | 0.6±0^**^ | 0.17±0.01^***^ | 0.72±0^**^ | 0.28±0.01^**^ | 0.11±0.01^**^ | 0.31±0.01^***^ | 0.1±0.01^**^ |
|  | *t*-FA100 |  | 0.4±0^*^ | 0.33±0.03^***^ | 0.17±0^***^ | 0.48±0.01^*^ | 0.05±0.04^**^ | 0.39±0.01^*^ | 0^*^ | 0.22±0.01^***^ | 0.08±0^*^ | 0.1±0.01^**^ |
| **Criollo** | Control | 14 | 1.12±0.02^***^ | 0.26±0.01^**^ | 0.06±0*** | 1.28±0.02^***^ | 0.3±0.01^***^ | 0.23±0.01^*^ | 0.03±0.02^***^ | 0^***^ | 0.01±0.02^***^ | 0^***^ |
|  | *p*-CA1 |  | 0.09±0^*^ | 0.22±0.02^**^ | 0.05±0.01^**^ | 0.39±0.01^*^ | 0^**^ | 0.28±0.02^**^ | 0^**^ | 0^***^ | 0.01±0.02^***^ | 0^***^ |
|  | *t*-FA1 |  | 0.12±0^**^ | 0.44±0.05^***^ | 0.02±0.01* | 1.18±0.08^**^ | 0^**^ | 0.56±0.03^***^ | 0.02±0.01^*****^ | 0^***^ | 0.02±0.01^***^ | 0^***^ |
| **Hass** | Control |  | 0.09±0.01^*^ | 0.57±0.04^***^ | 0.03±0^***^ | 0.69±0.03^***^ | 0^*^ | 0.4±0.01^**^ | 0.01±0.01^**^ | 0^***^ | 0.02±0.01^***^ | 0^***^ |
|  | *p*-CA1 |  | 0.11±0.01^**^ | 0.32±0.01^**^ | 0.02±0^**^ | 0.55±0.05^**^ | 0^*^ | 0.23±0.02^**^ | 0^**^ | 0^***^ | 0^*^ | 0^***^ |
|  | *p*-CA10 |  | 0.11±0.01^**^ | 0.1±0.01^*^ | 0.03±0^***^ | 0.24±0.02^*^ | 0.06±0^**^ | 0.55±0.03^**^ | 0.1±0.02^***^ | 0^***^ | 0.01±0^**^ | 0^***^ |
|  | *t*-FA1 |  | 0.34±0.01^***^ | 0.36±0.04^**^ | 0.01±0^*^ | 0.44±0.03^*^ | 0.1±0.01^***^ | 0.42±0.03^**^ | 0.02±0.01^**^ | 0^***^ | 0^*^ | 0^***^ |
|  | *t*-FA100 |  | 0^*^ | 0^*^ | 0^*^ | 0^*^ | 0^*^ | 7.06±3.63^***^ | 0^**^ | 0^***^ | 0^*^ | 0^***^ |
| **Criollo** | Control | 28 | 0.62±0.01^*^ | 0.25±0.01^**^ | 0.05±0.01^**^ | 0.96±0^***^ | 0^***^ | 0.72±0.01^***^ | 0.23±0.01^**^ | 0^***^ | 0.5±0^***^ | 0^***^ |
|  | *p*-CA1 |  | 3.56±0.06^***^ | 0^*^ | 0^*^ | 0.05±0.04^*^ | 0^***^ | 0^*^ | 0^*^ | 0^***^ | 0^*^ | 0^***^ |
|  | *t*-FA1 |  | 1.35±0.02^**^ | 0.49±0.03^***^ | 0.08±0^***^ | 0.74±0.01^**^ | 0.02±0.04^***^ | 0.12±0.10^**^ | 1.55±0.12^***^ | 0^***^ | 0.03±0^**^ | 0^***^ |
| **Hass** | Control |  | 0.4±0^*^ | 0.62±0.01^***^ | 0.05±0^**^ | 1.14±0.01^***^ | 0^***^ | 0.78±0.01^**^ | 0.04±0.01^***^ | 0^***^ | 0.01±0^***^ | 0^***^ |
|  | *p*-CA1 |  | 0.4±0.01^*^ | 0.36±0.01^**^ | 0.06±0^***^ | 0.75±0.01^*^ | 0^***^ | 1.3±0.04^**^ | 0.03±0.02^***^ | 0^***^ | 0^**^ | 0^***^ |
|  | *p*-CA10 |  | 1.36±0.03^***^ | 0^*^ | 0^*^ | 0.02±0.02^*^ | 0^***^ | 7.75±4.43^***^ | 0^*^ | 0^***^ | 0^**^ | 0^***^ |
|  | *t*-FA1 |  | 0.19±0.01^*^ | 0.24±0.01^*^ | 0.02±0.01^*^ | 0.85±0.02^**^ | 0^***^ | 0.08±0.01^**^ | 0.03±0.01^***^ | 0^***^ | 0^**^ | 0^***^ |
|  | *t*-FA100 |  | 1.12±0.01^**^ | 0^*^ | 0.03±0^*^ | 0.16±0.01^*^ | 0^***^ | 0.08±0^**^ | 0.01±0.01^**^ | 0^***^ | 0^**^ | 0^***^ |

†A general linear model (GLM) for metabolomics data was applied with default settings to determine the statistical significance between treatments

‡Data are mean ± standard deviation reported in mµ/g of dried PEM´s

§ *, **, *** represent a significant difference among treatments (*P* < 0.05)

¶Concentration below the limit of quantification is expressed as

**Table S3.** Putative identification of compounds detected in embryogenic Criollo and Hass cultures treated with *P*-coumaric acid (1 µM) and *trans*-ferulic acid (100 µM) after 12 h and 28 d.

| Cultivar | Time | R_t_ | -FooDB ID | Chemical formula | Ion | Error  (<5 ppm) | Putative candidate |
| --- | --- | --- | --- | --- | --- | --- | --- |
| Hass  *t*-FA100 | 12 h | 4.41 | FDB020703 | C_19_H_28_O_10_ | M+H | 3 | Disaccharide |
|  |  | 1.3 | FDB020638 | C_10_H_20_O_7_ | M+Na | 1 | Disaccharide |
|  |  | 4.13 | FDB000825 | C_16_H_22_O_9_ | M+H | 2 | Disaccharide |
|  |  | 3.69 | FDB014518 | C_12_H_14_N_10_O_2_ | M+H | 3 | 1-Methylguanine |
|  |  | 0.46 | FDB001128 | C_6_H_12_O_6_ | M+Na | 3 | Hexose |
|  |  | 7.53 | FDB017851 | C_18_H_32_O_5_ | M+Na | 1 | Auxin a |
|  |  | 1.15 | FDB003554 | C_10_H_14_N_5_O_4_^+^ | [M+H]^+^ | -3 | Adenosine |
|  | 28 d | 0.5 | FDB001128 | C_6_H_12_O_6_ | M+Na | 2 | Hexose |
|  |  | 1.14 | FDB001182 | C_12_H_22_O_11_ | M+Na | 0 | Cellulose |
|  |  | 8.77 | FDB023381 | C_18_H_39_NO_3_ | M+H | 0 | Phytosphingosine |
|  |  | 1.18 | FDB003554 | C_10_H_14_N_5_O_4_^+^ | [M+H]^+^ | 0.7 | Adenosine |
| Criollo  *p*-CA1 | 12 h | 0.77 | FDB030763 | C_10_H_12_ N_5_O_8_P | M+H | 2 | cyclic-AMP |
|  |  | 1.19 | FDB004222 | C_5_H_5_N_5_O | M+H | 1 | Guanine |
|  |  | 5.42 | FDB017851 | C_18_H_32_O_5_ | M+Na | 1 | Auxin a |
|  |  | 4.284 | FDB011252 | C_16_H_30_O_10_ | M+Na | 2 | Trans-1,2,10-Trihydroxydihydrolinalyl oxide 7-glucoside |
|  |  | 1.77 | FDB022314 | C_14_H_18_O_8_N_5_^+^ | [M+H]^+^ | -2.9 | Succinoadenosine |
|  |  | 8.67 | FDB002683 | C_46_H_71_O_8_Na^+2^ | [M+H+Na]^+2^ |  | 1,26-hexacosanediol diferulate |
|  |  | 0.5 |  | C_12_H_22_O_11_Na^+^ | [M+Na]^+^ | -1.9 | Disaccharide |
|  | 28 d | 8.72 | FDB002683 | C_46_H_72_O_8_^+2^ | [M+2H]^+2^ | -6.5 | 1,26-hexacosanediol diferulate |
|  |  | 0.48 |  | C_12_H_22_O_11_K^+^ | [M+K]^+^ | 2 | Disaccharide |
|  |  | 8.73 | FDB002683 | C_46_H_71_O_8_Na^+2^ | [M+H+Na]^+2^ | 6.5 | 1,26-hexacosanediol diferulate |
|  |  | 1.18 | FDB003554 | C_10_H_14_N_5_O_4_^+^ | [M+H]^+^ | 0.7 | Adenosine |

Table S4. Primers designed for quantification of relative expression by qPCR.

| Oligonucleotides for quantitative TaqMan™ RT-PCR | | |
| --- | --- | --- |
| Rubisco | AY337727 | Probe  5’/56-FAM/TGAATGTCT/ZEN/CCG TGG TGG ACT TGA /3IABkFQ/3’  Forward  5’-GGGTTATCCGCCAAGAACTAC-3’  Reverse  5’-CGGTCTCTCCAACGCATAAA-3’ |
| Chalcone-flavanone isomerase family | AT5G05270 | Probe  5’/56-FAM/TGC TTC AGT /ZEN/GTACAATCCAACGGCT/3IABkFQ/3’  Forward  5’-GGTCAACAGTACTCAGAGAAGG-3’  Reverse  5’-GAACTTTTCAACGGCTATGGC-3’ |
|  |  | Probe  5’/56-FAM/CAAGCGATC/ZEN/CCTCACTGCACTCT3IABkFQ/3’  Forward  5’-AGGTTCTCAATATGGCGTTCAG-3’  Reverse  5’-TCAATGAGTTTCTCCAGCGC-3’ |
| Flavanone 3-hydroxylase | AT3G51240 | Probe  5’/56-FAM/ATGTCTGGA/ZEN/GGGAAG AAG GGA GGT /3IABkFQ/  Forward  5’-GCTGAAGAGAAGCTGCGATATG-3’  Reverse  5’-GGGTAGGAGAAGAAGGTTACGA-3 |
| Phenylalanine ammonia-lyase 1 | AT2G37040 | Probe  CAAGCGATCCCTCACTGCACTCT  /56-FAM/CGC TGA GAA /ZEN/CAC CTT GTC GAA CTC C/3IABkFQ/  Forward  5’-GCTGACGGGTGAGAAAGTC-3’  Reverse  5’-CAATTACCTTCCCTTGGCAAATC-3’ |
